# Organophosphorus pesticide chlorpyrifos intake promotes obesity and insulin resistance through impacting gut and gut microbiota

**DOI:** 10.1186/s40168-019-0635-4

**Published:** 2019-02-11

**Authors:** Yiran Liang, Jing Zhan, Donghui Liu, Mai Luo, Jiajun Han, Xueke Liu, Chang Liu, Zheng Cheng, Zhiqiang Zhou, Peng Wang

**Affiliations:** 1 Beijing Advanced Innovation Center for Food Nutrition and Human Health, College of Science, China Agricultural University, No. 2, West Yuanmingyuan Road, Beijing, 100193 People’s Republic of China; 2 College of Chemistry and Biological Engineering, University of Science and Technology Beijing, No. 30, Xueyuan Road, Beijing, 100083 People’s Republic of China

**Keywords:** Obesity, Gut microbiota, Organophosphorus pesticide, Chlorpyrifos, Lipopolysaccharide, Insulin resistance

## Abstract

**Background:**

Disruption of the gut microbiota homeostasis may induce low-grade inflammation leading to obesity-associated diseases. A major protective mechanism is to use the multi-layered mucus structures to keep a safe distance between gut epithelial cells and microbiota. To investigate whether pesticides would induce insulin resistance/obesity through interfering with mucus-bacterial interactions, we conducted a study to determine how long-term exposure to chlorpyrifos affected C57Bl/6 and CD-1 (ICR) mice fed high- or normal-fat diets. To further investigate the effects of chlorpyrifos-altered microbiota, antibiotic treatment and microbiota transplantation experiments were conducted.

**Results:**

The results showed that chlorpyrifos caused broken integrity of the gut barrier, leading to increased lipopolysaccharide entry into the body and finally low-grade inflammation, while genetic background and diet pattern have limited influence on the chlorpyrifos-induced results. Moreover, the mice given chlorpyrifos-altered microbiota had gained more fat and lower insulin sensitivity.

**Conclusions:**

Our results suggest that widespread use of pesticides may contribute to the worldwide epidemic of inflammation-related diseases.

**Electronic supplementary material:**

The online version of this article (10.1186/s40168-019-0635-4) contains supplementary material, which is available to authorized users.

## Background

The global epidemic of obesity has rapidly increased with economic development and changes in dietary patterns. The incidence of obesity is 10.7% in China, 12.8% in the European Union, and 30.4% in the USA [1–3]. Epidemiologic studies have shown that obesity not only causes excessive deposition of fat in the body, but also increases the risk of developing chronic diseases such as type 2 diabetes (T2D) and cardiovascular disease [4]. The development of obesity is complex and is thought to involve both genetic and environmental factors as well as their interaction. Insulin resistance (IR) is commonly seen in obese individuals and plays a key role in the development of T2D [5]. Recent studies have demonstrated that obesity is not simply an excessive accumulation of fat but is also associated with a low-grade chronic inflammatory state, which is the main factor that induces IR. One of the molecular mechanisms underlying IR development is increased expression of pro-inflammatory cytokines in the process of low-grade inflammation [6, 7]. Pro-inflammatory cytokines may interfere with the insulin signaling pathway to cause IR in peripheral tissues, resulting in the dysregulated metabolism of carbohydrates and lipids [8]. Increasing evidence suggests that the gut microbiota plays an important role in the development of low-grade inflammation [9].

The human gut contains numerous microorganisms that comprise a large and dynamic ecosystem. Gut microbiota influences the host in multiple aspects including provision of nutrients, modulation of metabolism, and regulation of immunity. On the other hand, disruption of the health and balance of the gut microbiota may induce low-grade inflammation, leading to obesity-associated diseases [10–12]. Microbiota-induced low-grade inflammation is mainly induced by lipopolysaccharides (LPS), which are present in the cell walls of Gram-negative bacteria. After gaining entry into the body, LPS stimulate the production of several pro-inflammatory cytokines to induce low-grade inflammation by binding to the Toll-like receptor 4 (TLR-4) on the surface of innate immune cells. Increased plasma levels of LPS are sufficient to trigger IR and obesity [13, 14]. Increased entry of LPS into the body is mainly caused by two factors: disruption of the microbiota balance, which increases the LPS-bearing bacteria population, directly elevating LPS levels in the gut; and the broken integrity of the gut barrier, which allows LPS to more easily enter the body. Therefore, all exogenous compounds that can disrupt the microbiota balance and increase gut permeability are potential risk factors for inducing low-grade inflammation. Pesticides are a type of exogenous compound that people are commonly exposed to; they are thought to significantly impact obesity [15] as well as affect gut microbiota and gut barrier function [16, 17]. However, since this has not been confirmed, it is important to investigate the effects of pesticides on gut microbiota and obesity.

Organophosphorus (OP) pesticides have been widely used since the late nineteenth century and early twentieth century, and even today, are still among the most commonly used type of pesticides due to their ideal bioactivity. Epidemiologic studies have shown that the extensive use of OP pesticides is an important risk factor for developing metabolic diseases [18, 19]. Chlorpyrifos is one of the most widely used OP pesticides around the world which makes people to be frequently exposed to it. Recent studies showed that chlorpyrifos was frequently detected in food, with the highest rate of 38.3% [20, 21]. Moreover, the highest dietary exposure of chlorpyrifos was 4 μg/kg per day in the residents of Greater Baltimore, USA [22]. According to European Food Safety Authority (EFSA), chlorpyrifos was one of the pesticides that were most frequently exceeded the acute reference dose (ARfD) in food products [23]. Recent studies have found that animals exposed to chlorpyrifos can develop hyperlipidemia, hyperinsulinemia, and obesity [24, 25]. In addition, in vivo and in vitro studies have demonstrated that chlorpyrifos can impair the intestinal epithelial cell zonula occludens-1 (ZO-1), a tight junction-associated protein, resulting in increased intestinal permeability [17, 26]. Based on these results, we hypothesized that chlorpyrifos-induced obesity may be mediated through increased intestinal permeability or altered microbiota, either of which can facilitate the increased entry of LPS into the body to cause low-grade inflammation, ultimately leading to IR and obesity. Both dietary patterns and genetic background have enormous impacts on the occurrence of obesity and IR. Two commercial mice chow containing 10% and 60% lipids are widely used to simulate different dietary patterns. Inbred strain C57Bl/6 mice are genetically similar and facilitate reproducible data generation. Outbred strain CD-1 (ICR) mice are nonhomogeneous populations with high genotypic and phenotypic variance, which supposedly more accurately mimic what one would find in humans. Both of these strains of mice are widely used together to study genetic influences.

The main goal of this study was to identify new mechanisms by which pesticides affect humans, so that the information obtained can be used for more comprehensive assessment of the chronic health risks of pesticide exposure. To this end, we fed C57Bl/6 and CD-1 (ICR) mice a high-fat diet (HFD) or normal-fat diet (NFD) and investigated if chlorpyrifos could induce IR and obesity through the above-mentioned pathways. The results showed that chlorpyrifos altered the microbiota composition and compromised the integrity of the gut barrier, which induced IR and obesity by upregulating inflammatory pathways. Moreover, chlorpyrifos-altered microbiota could affect the occurrence of obesity and impaired insulin sensitivity.

## Results

### Effects of chlorpyrifos on body weight and food intake

**Fig. 1:**
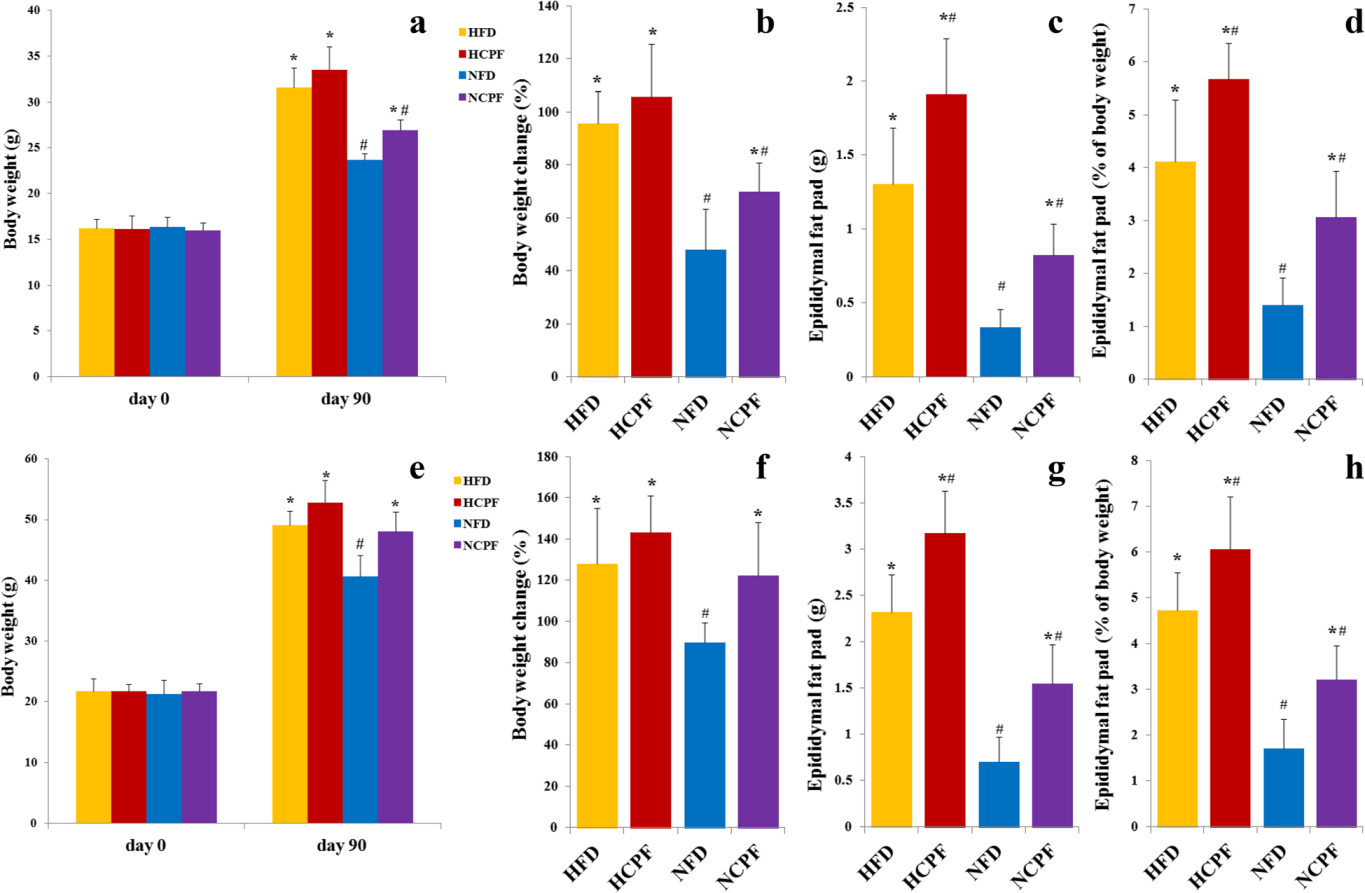
Effects of chlorpyrifos administration on body composition. C57Bl/6 (**a**–**d**) and CD-1 (ICR) (**e**–**h**) mice were fed either a normal-fat diet (NFD) or high-fat diet (HFD) for 12 weeks. NCPF and HCPF mice were treated daily with 5 mg/kg chlorpyrifos. NFD and HFD control mice were gavaged with vehicle (corn oil). Effects of chlorpyrifos treatment on body weight (**a**, **e**), percent of body weight change (**b**, **f**), epididymal fat pad weight (**c**, **g**), and epididymal fat pad content of whole body weight (**d**, **h**) were measured (*n* = 8). Data are expressed as the mean ± SEM.*P < 0.05 vs. NFD group; ^#^*P* < 0.05 vs. HFD group. NFD normal-fat diet, NCPF normal-fat diet + chlorpyrifos, HFD high-fat diet, HCPF high-fat diet + chlorpyrifos

Chronic exposure to chlorpyrifos enhanced weight gain in both C57Bl/6 and CD-1 (ICR) mice (Fig. 1). The weight and percent of body weight change (expressed as percent of initial body weight) were significantly higher in the normal-fat diet + chlorpyrifos (NCPF) group compared with the NFD group, but was not significantly different between the high-fat diet + chlorpyrifos (HCPF) and HFD groups (Fig. 1a, b, e, and f). The results of epididymal fat pad weight reflected the changes in body weight (i.e., in both HFD and NFD groups, the absolute epididymal fat pad weight (Fig. 1c, g) and epididymal fat pad content of whole body weight (%, Fig. 1d, h) were consistently higher in the treatment groups than in the control groups). HFD groups also had a higher epididymal fat pad weight and epididymal fat pad content of whole body weight (%) than the NFD groups. Food intake was not different in NFD-fed (NFD and NCPF) and HFD-fed (HFD and HCPF) groups in both C57Bl/6 and CD-1 (ICR) mice (Additional file 1: Figure S1 a, e), indicating that the effects of chlorpyrifos on food intake were not the reason for chlorpyrifos-induced weight gain in mice. The above results revealed that chlorpyrifos treatment could increase body weight in NFD-fed mice, and epididymal fat pad weight and epididymal fat pad content in both HFD and NFD-fed mice, but have limited effects on food intake.

### Effects of chlorpyrifos on IR

**Fig. 2:**
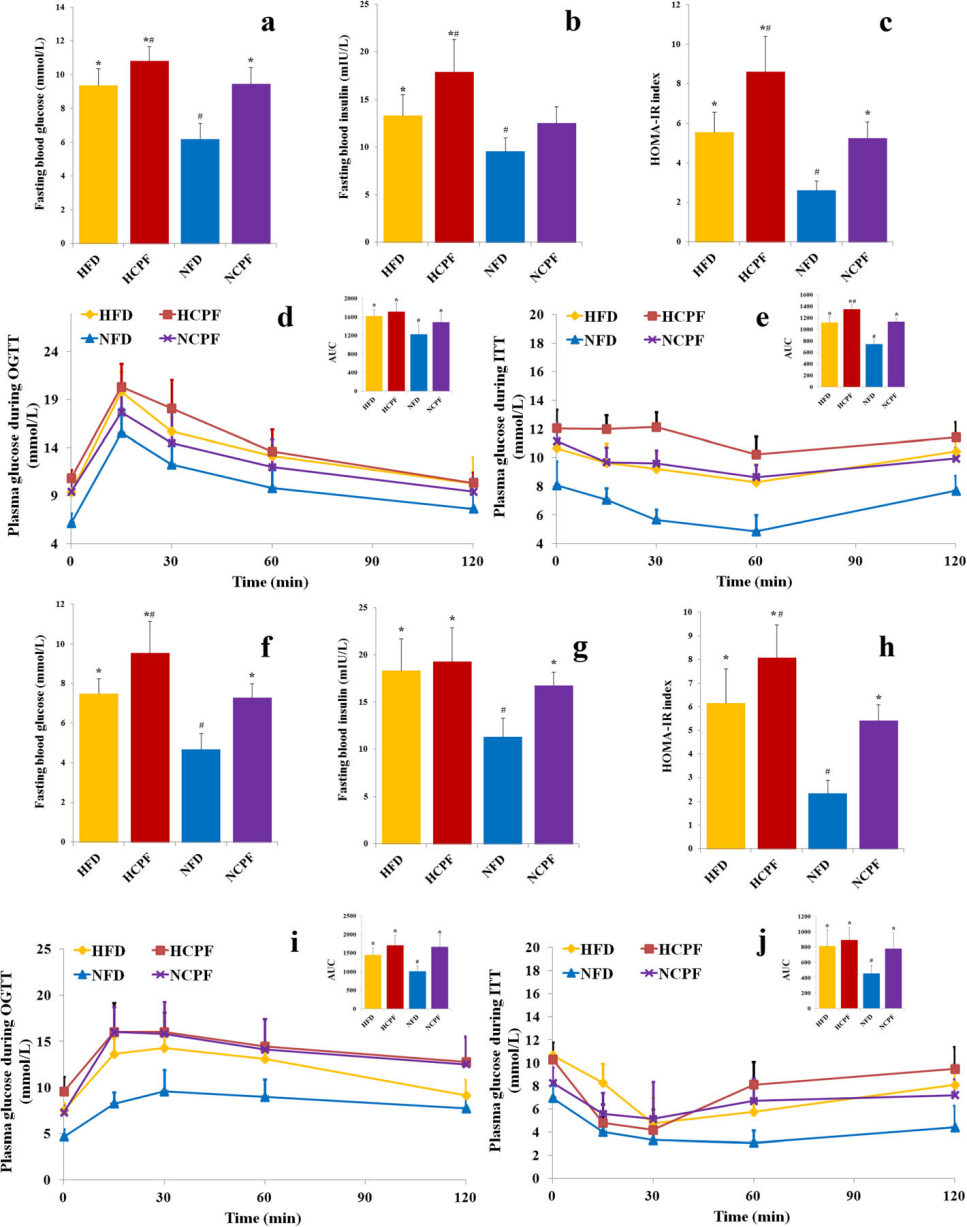
Effects of chlorpyrifos administration on fasting glucose, fasting insulin, glucose tolerance, and insulin sensitivity in C57bl/6 (**a**–**e**) and CD-1 (ICR) mice (**f**–**j**). Mice were deprived of food for 12 h and 6 h to determine fasting glucose (**a**, **f**) and insulin (**b**, **g**). HOMA-IR index was calculated based on fasting glucose and insulin (**c**, **h**). Mice were fasted for 12 h, and an oral glucose tolerance test (**d**, **i**) was performed. Mice were fasted for 6 h, and an insulin tolerance test (**e**, **j**) was performed (*n* = 8). Data are expressed as the mean ± SEM. *P < 0.05 vs. NFD group; ^#^*P* < 0.05 vs. HFD group. NFD normal-fat diet, NCPF normal-fat diet + chlorpyrifos, HFD high-fat diet, HCPF high-fat diet + chlorpyrifos, HOMA-IR homeostasis model assessment of insulin resistance, OGTT oral glucose tolerance test, ITT insulin tolerance test

It is commonly believed that IR is an early indicator of T2D and obesity [27]. Given this premise, to better understand the harmful effects of chlorpyrifos on human health, the effects of chlorpyrifos treatment on glucose homeostasis and insulin sensitivity were determined (Fig. 2). The results (Fig. 2a, b, f, and g) showed that in both the HFD and NFD groups, chlorpyrifos treatment led to significantly higher concentrations of fasting blood glucose and insulin compared to the corresponding control groups (except fasting insulin in NFD-fed C57Bl/6 and HFD-fed CD-1 (ICR) mice). These results suggested that chlorpyrifos may impair the insulin sensitivity of these mice, which was further supported by the higher homeostasis model assessment of insulin resistance (HOMA-IR) index observed in the chlorpyrifos-treated mice (Fig. 2c, h). NCPF mice had significantly lower insulin sensitivity and glucose tolerance in comparison with NFD controls as assessed by oral glucose tolerance test (OGTT) and insulin tolerance test (ITT) in both C57Bl/6 (Fig. 2d, e) and CD-1 (ICR) mice (Fig. 2i, j). In addition, compared to those in HFD control groups, HCPF C57Bl/6 mice had significantly lower insulin sensitivity. In these two strains of mice, the HFD groups had significantly higher fasting blood glucose and insulin, HOMA-IR index, and lower insulin sensitivity and glucose tolerance than the NFD groups. These data indicated that chlorpyrifos treatment could impair glucose homeostasis and induce insulin resistance in both HFD- and NFD-fed mice.

### Effects of chlorpyrifos on gut permeability

**Fig. 3:**
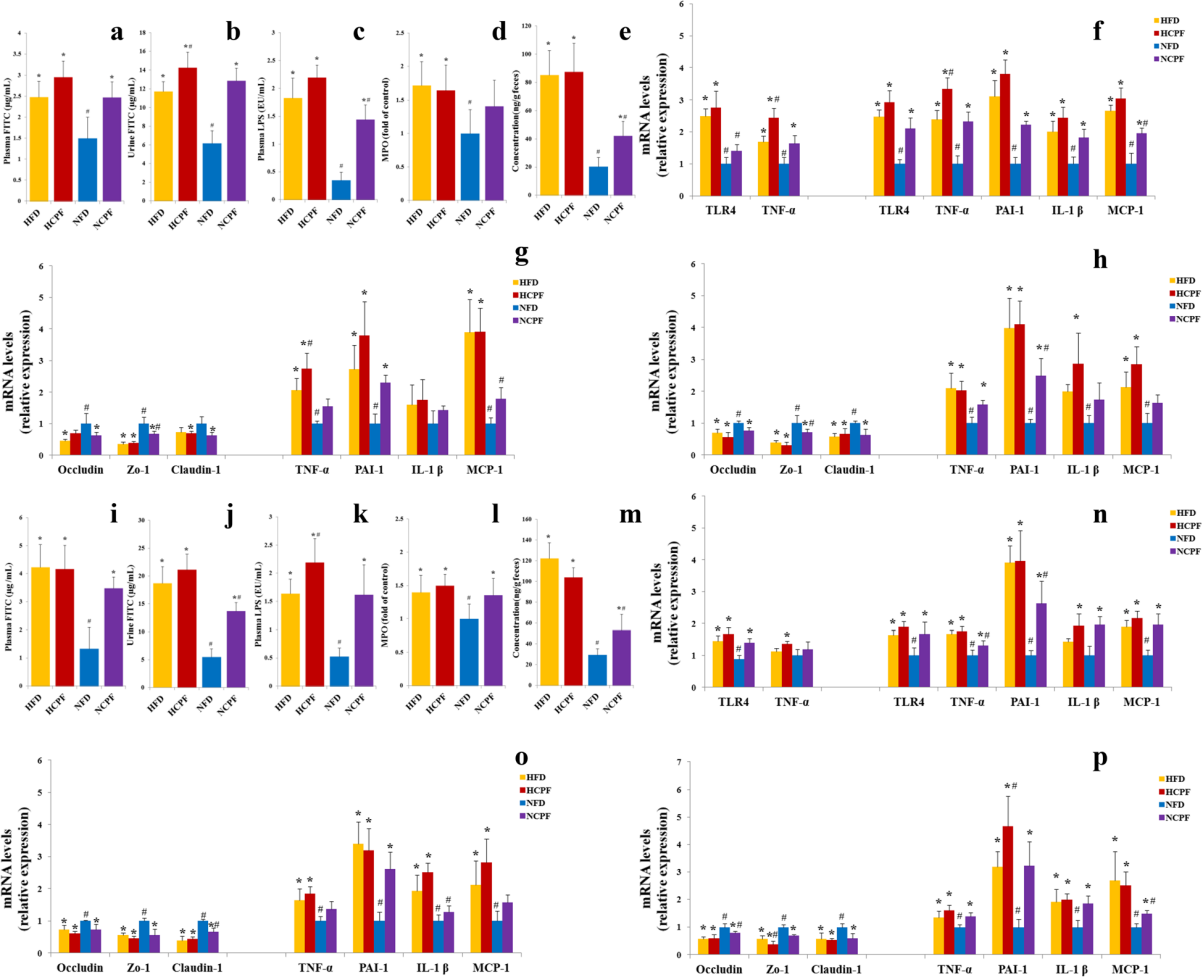
Effects of chlorpyrifos administration on intestinal permeability (**a**, **b** and **i**, **j**, *n* = 8), circulating LPS (**c**, **k**, *n* = 8), MPO activity (**d**, **l**, *n* = 8), fecal lipocaline-2 levels (**e**, **m**, *n* = 8), inflammation in liver and epididymal fat (**f**, **n**, *n* = 5), and tight junction proteins expression and inflammation in ileum and colon (**g**, **h** and **o**, **p**, *n* = 5) in C57Bl/6 (**a**–**h**) and CD-1 (ICR) mice (**i**–**p**). Data are expressed as the mean ± SEM. *P < 0.05 vs. NFD group; ^#^*P* < 0.05 vs. HFD group. NFD normal-fat diet, NCPF normal-fat diet + chlorpyrifos, HFD high-fat diet, HCPF high-fat diet + chlorpyrifos

Previous studies have found that IR is often associated with low-grade inflammation, and the latter is also associated with increased gut permeability [13]. In the current study, the effects of chlorpyrifos on gut permeability and mRNA expression of tight junction proteins (ileum and colon) were determined. In the gut permeability assay, after the administration of FITC-labeled dextran to mice, plasma and urine FITC-dextran levels were significantly higher in chlorpyrifos-treated C57Bl/6 and CD-1 (ICR) mice fed a normal-fat diet compared to those in the control groups (Fig. 3a, b, i, and j). In addition, urine FITC-dextran levels were also significantly higher in chlorpyrifos-treated C57Bl/6 mice fed a high-fat diet than those in the control groups (Fig. 3b, j). These results indicated that chlorpyrifos could increase gut permeability. In addition, chlorpyrifos significantly decreased the mRNA expression of tight junction proteins (occludin, claudin 1, and ZO-1) in ileum and colon in NFD-fed groups (Fig. 3g, h, o, and p). Because gut permeability is controlled by these specific tight junction proteins, chlorpyrifos may increase intestinal permeability by reducing the expression of tight junction proteins. Since increased gut permeability often causes elevated plasma LPS levels, the plasma LPS level was further examined and the results showed that chlorpyrifos-treated mice had higher plasma LPS in C57Bl/6 fed a normal-fat diet and CD-1 (ICR) mice fed a high-fat diet or normal-fat diet compared to the corresponding control mice (Fig. 3c, k). Compared with NFD-fed groups, HFD-fed groups had significantly higher intestinal permeability and plasma LPS concentration in both C57Bl/6 and CD-1 (ICR) mice. The above results suggested that chlorpyrifos treatment could increase gut permeability in NFD-fed mice, leading to increased plasma LPS levels.

### Chlorpyrifos induces pro-inflammatory response

High circulating LPS levels are associated with obesity and IR [28, 29]. LPS can bind and activate TLR-4 to increase the expression of pro-inflammatory mediators such as TNF-α, and these pro-inflammatory mediators can interfere with the binding of insulin to its receptor, leading to IR and obesity [28, 30]. To examine whether TLR-4 pathway is involved in chlorpyrifos-induced IR and obesity, the TLR-4 expression in liver and adipose tissues was examined (Fig. 3f, n). The results showed that in both C57Bl/6 and CD-1 (ICR) mice fed a NFD, chlorpyrifos-treated mice had higher TLR-4 expression in the fat pad and liver, suggesting that chlorpyrifos-induced increases of LPS can upregulate TLR-4 expression. To further investigate whether chlorpyrifos could cause low-grade inflammation through the LPS pathway, expression of the major pro-inflammatory mediators involved in IR and obesity in the liver (TNF-α) and adipose tissue (TNF-α, MCP-1, IL-1 β, PAI-1) was examined (Fig. 3f, n). It was found that chlorpyrifos could upregulate the expression of these pro-inflammatory mediators in the liver and adipose tissues in NFD-fed groups. Similar results were also found in the HFD groups compared with the NFD groups in both C57Bl/6 and CD-1 (ICR) mice. The intestine could express inflammatory mediators, release inflammatory mediators into blood, and then increase inflammation in tissues. In this case, the effects of chlorpyrifos on intestinal inflammation were detected. Colon length, MPO activity, and fecal lipocaline-2 levels were detected as the indicators of gut inflammation, and the results showed that treatment with chlorpyrifos did not significantly shorten the colon length (Additional file 1: Figure S1b, f), but could upregulate MPO activity (Fig. 3d, l) and fecal lipocaline-2 levels (Fig. 3e, m) in NFD-fed groups, suggesting that chlorpyrifos may induce gut inflammation. Proinflammatory cytokines expression (TNF-α, MCP-1, IL-1 β, PAI-1) in the ileum and colon and the concentration of proinflammatory cytokines in plasma were also measured (Fig. 3g, h, o, and p and Additional file 2: Figure S2). The results showed that chlorpyrifos increased these pro-inflammatory mediators in both C57Bl/6 and CD-1 (ICR) mice, especially in NFD-fed groups. These data indicated that chlorpyrifos treatment could induce pro-inflammatory response in mice.

### Effects of chlorpyrifos on the gut microbiota

**Fig. 4:**
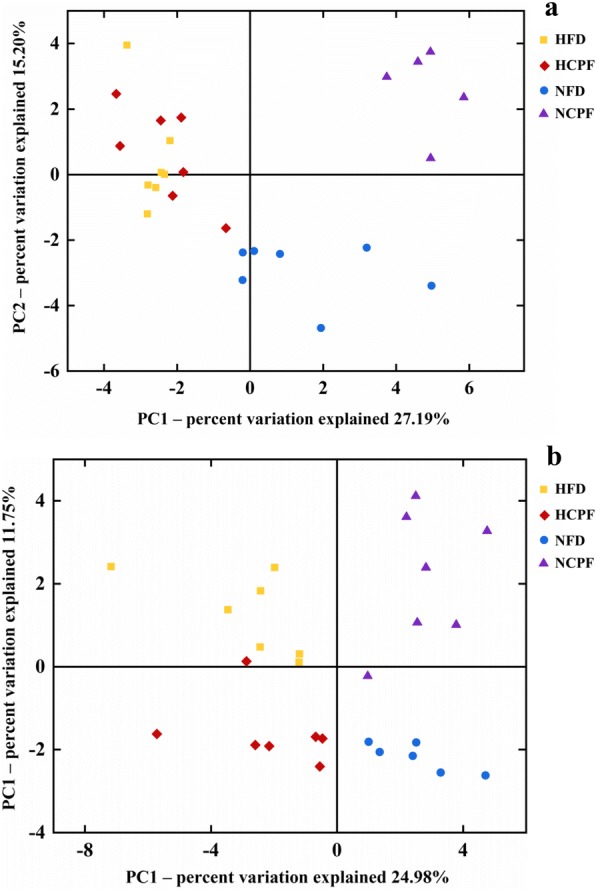
Principal coordinates analysis (PCA) of the gut microbiota metagenomes (**a** for C57Bl/6 and **b** for CD-1(ICR)). The PCA analysis focus on grouping sampled fecal communities with respect to diet and treatment. NFD normal-fat diet, NCPF normal-fat diet + chlorpyrifos, HFD high-fat diet, HCPF high-fat diet + chlorpyrifos

**Fig. 5:**
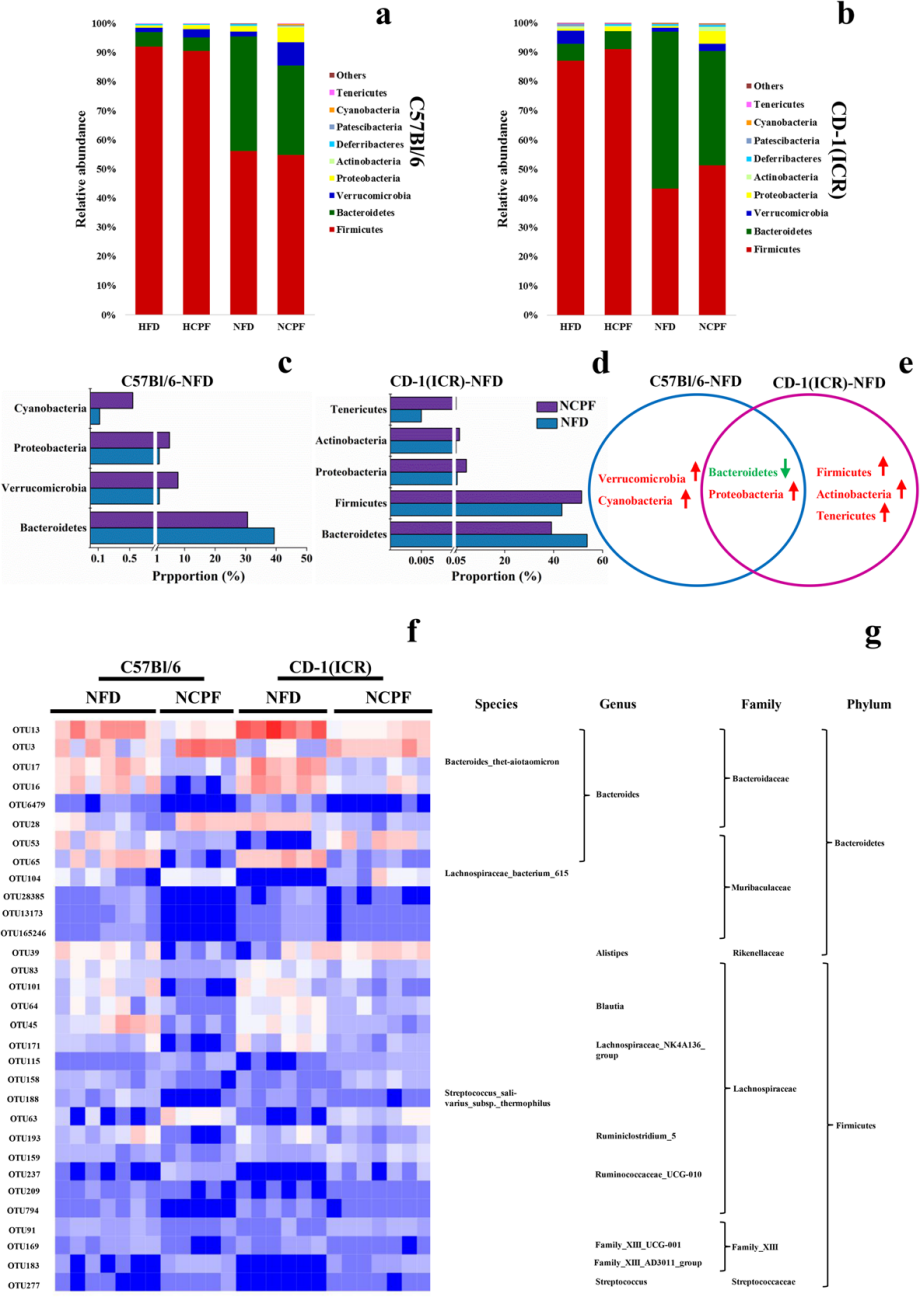
Microbiota composition of NFD- and HFD-fed mice treated with or without chlorpyrifos (*n* = 5 for NCPF of C57Bl/6 mice, *n* = 6 for NFD of CD-1 (ICR) mice, *n* = 7 for the rest). Relative abundance distribution of bacterial phyla from fecal metagenomes of NFD, NCPF, HFD, and HCPF mice at week 12 (**a**, **b**). Statistical comparisons of gut metagenomic profiles at phyla level of NFD and NCPF mice (**c**, **d**, and **e**). Only features (phyla) with a *P* value of < 0.05 were shown. Heatmap showing the abundance of 31 OTUs was significantly altered by chlorpyrifos in both NFD-fed C57BL/6 and CD-1 (ICR) mice (*P* < 0.05), blue and red signify underrepresented and overrepresented respectively (**f**, **g**). Data are expressed as the mean ± SEM. NFD normal-fat diet, NCPF normal-fat diet + chlorpyrifos, HFD high-fat diet, HCPF high-fat diet + chlorpyrifos

The gut microbiota plays an important role in the development of obesity, and a number of studies have shown that exposure to certain exogenous compounds may cause alterations in its composition to either enhance or mitigate obesity [31, 32]. Therefore, in the current study, gut microbiota in mice was examined and analyzed to determine chlorpyrifos-induced change in gut microbiota composition (*n* = 5 for NCPF of C57Bl/6 mice, *n* = 6 for NFD of CD-1 (ICR) mice, *n* = 7 for the rest). The results showed that chlorpyrifos treatment did not have significant effects on the absolute abundance of total fecal bacteria (Additional file 1: Figure S1 c, g). The degree of bacterial taxonomic similarity between metagenomics samples at the genus level was analyzed to assess the composition of bacterial community in the different groups (Additional file 3: Figure S3 and Additional file 4: Figure S4). Principal component analysis (PCA) was used to reveal clustering of the bacterial communities based on the OTUs (Fig. 4). The results showed that in both C57Bl/6 and CD-1 (ICR) mice, the gut microbiota composition in the NFD and HFD groups and in the NFD and NCPF groups could be discriminated by PCA, but not in the HFD and HCPF groups. Thus, the following analysis was focused on the effects of chlorpyrifos on microbiota composition in mice fed NFD. In both C57Bl/6 and CD-1 (ICR) mice fed NFD, chlorpyrifos treatment resulted in an increase in *Proteobacteria* phyla and a decrease in *Bacteroidetes* phyla (Fig. 5a–e), which indicated that these two phyla were the primary gut microbiota that were affected by chlorpyrifos in mice. In the further analysis of the common changes observed in both strains of mice in the OTUs induced by chlorpyrifos treatment, it was found that compared with the control mice, chlorpyrifos-treated mice had affected *Bacteroidaceae*, *Muribaculaceae*, and *Rikenellaceae*, *Lachnospiraceae*, *Family_XIII*, and *Streptococcaceae* in both C57Bl/6 and CD-1 (ICR) mice (Fig. 5f, g and Additional file 3: Figure S3). The changes in common species of gut bacteria observed in both C57Bl/6 and CD-1 (ICR) mice indicated that these are core microflora of the mouse gut microbiota impacted by chlorpyrifos. Fecal LPS levels were detected, and the results showed that chlorpyrifos treatment did not increase fecal LPS levels significantly (Additional file 1: Figure S1 d, h). The above results revealed that chlorpyrifos treatment could impact mice’s microbiota composition, but have limited effects on fecal LPS levels.

### Effects of chlorpyrifos-derived gut microbiota on mice

**Fig. 6:**
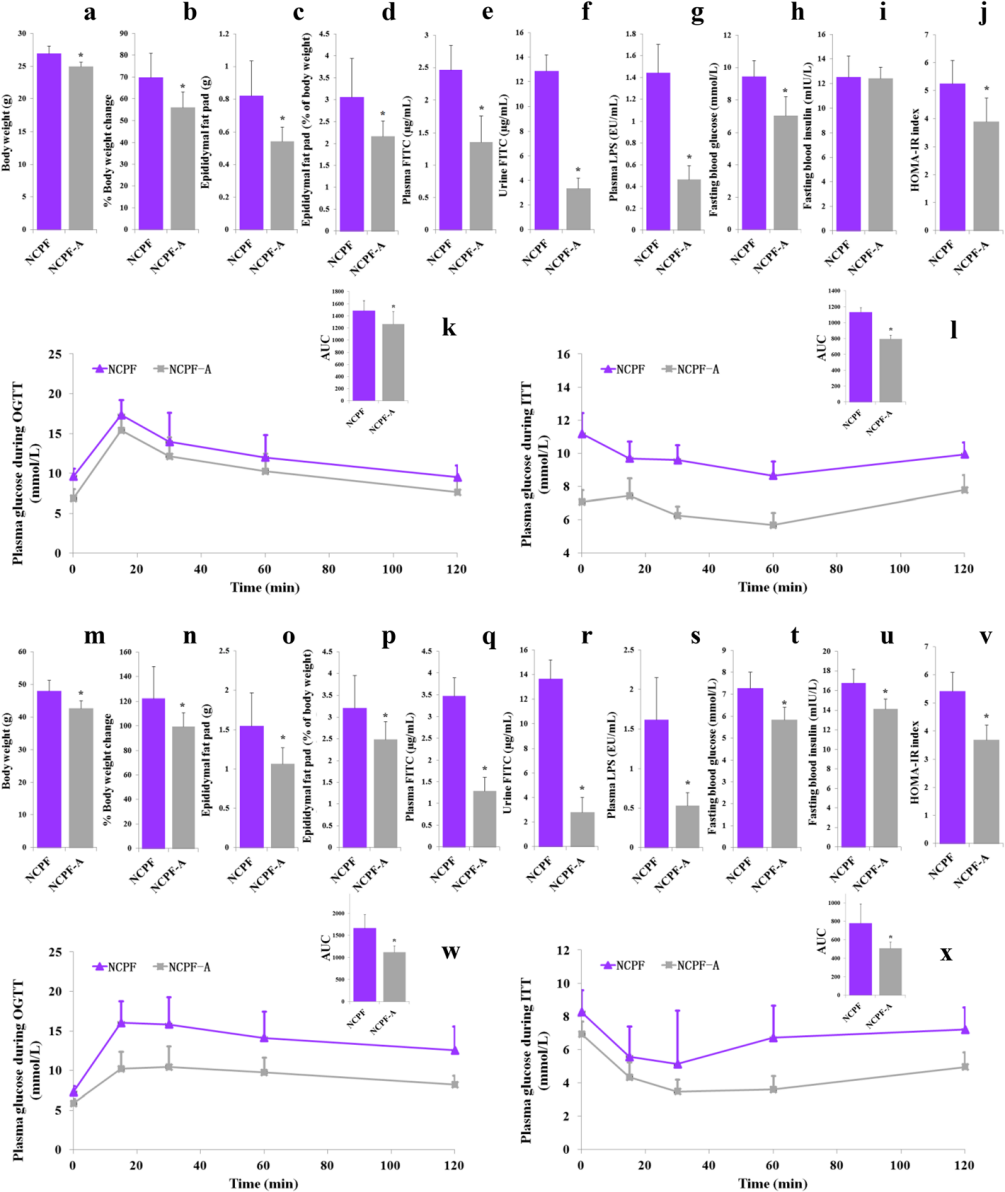
Effects of antibiotic treatment on body weight (**a**, **m**), changed body weight (%, **b**, **n**), epididymal fat pad weight (**c**, **o**), epididymal fat pad content of whole body weight (**d**, **p**), plasma FITC level (**e**, **q**), urine FITC level (**f**, **r**), plasma LPS (**g**, **s**), fasting blood glucose (**h**, **t**), fasting blood insulin (**i**, **u**), HOMA-IR index (**j**, **v**), glucose tolerance (**k**, **w**), and insulin sensitivity (**l**, **x**) in NCPF-fed C57Bl/6 (**a**–**l**) and CD-1 (ICR) mice (**m**–**x**). Data are expressed as the mean ± SEM. *P < 0.05. NCPF normal-fat diet + chlorpyrifos, NCPF-A normal-fat diet + chlorpyrifos + antibiotic

Previous studies have shown that xenobiotics exposure could impact gut microbiota composition and sometimes the altered microbiota alone could affect the occurrence of obesity and insulin resistance [10]. In the current study, the role of chlorpyrifos-altered microbiota was investigated by treating NCPF group mice with antibiotic (assigned as NCPF-A) according to the previous study [28]. The antibiotics used here were ampicillin and neomycin, which are broad-spectrum antibiotics that are poorly absorbed without any systemic effects [33]. As shown in Fig. 6, after 4 weeks’ antibiotic treatment, NCPF-A group had lower body weight, percent of body weight change, epididymal fat pad weight, epididymal fat pad content of whole body weight (%), plasma FITC level, urine FITC level, plasma LPS, fasting blood glucose, and HOMA-IR index and higher insulin sensitivity (assessed by ITT) and glucose tolerance (assessed by OGTT) than NCPF group in both C57Bl/6 and CD-1 (ICR) mice.

**Fig. 7:**
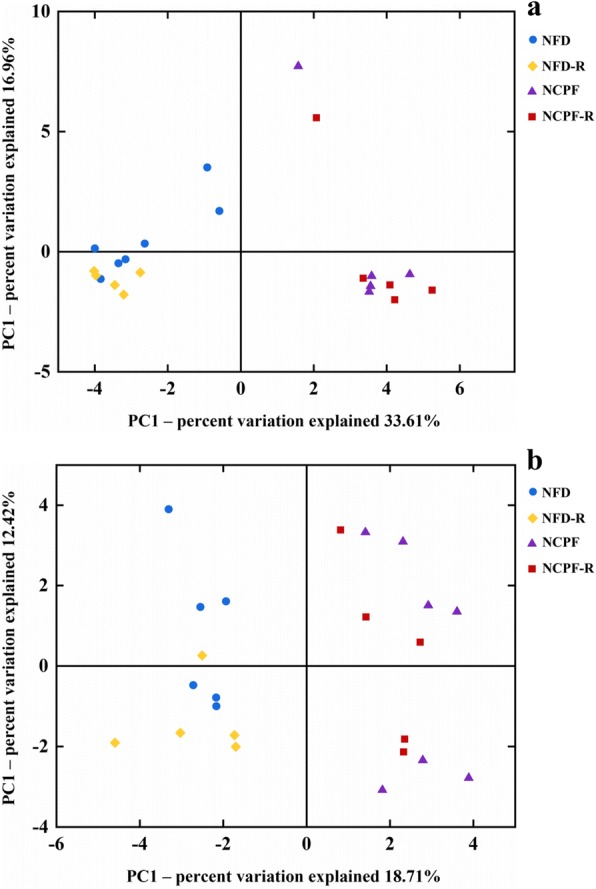
Principal coordinates analysis (PCA) of the gut microbiota metagenomes from NFD-R. NDF, NCPF-R, and NCPF groups (**a** for C57Bl/6 and **b** for CD-1(ICR)). NFD normal-fat diet, NCPF normal-fat diet + chlorpyrifos, NFD-R re-colonized with NFD group’s microbiota, NCPF-R re-colonized with NCPF group’s microbiota

**Fig. 8:**
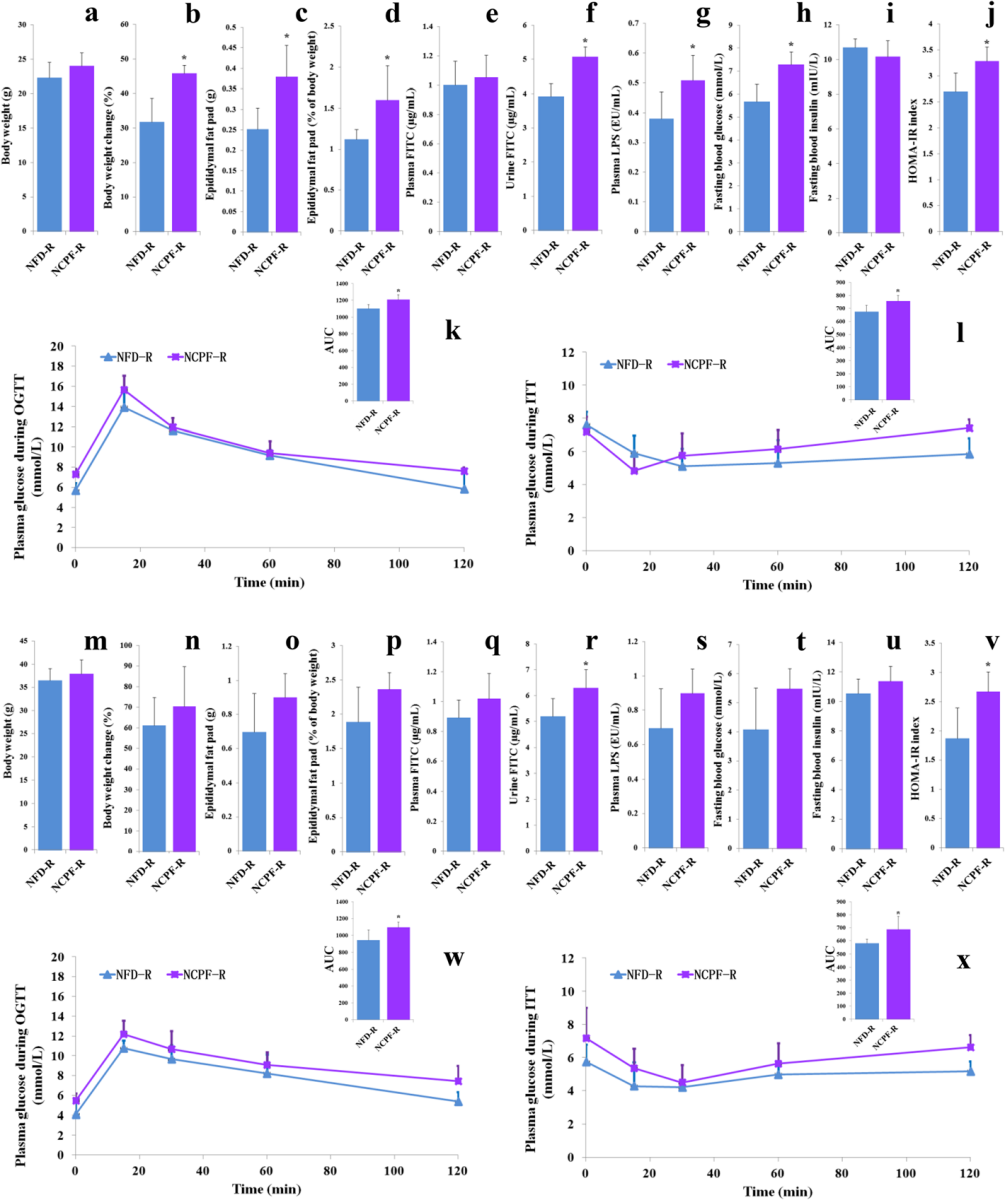
Effects of chlorpyrifos-altered microbiota on body weight (**a**, **m**), percent of body weight change (**b**, **n**), epididymal fat pad weight (**c**, **o**), epididymal fat pad content of whole body weight (**d**, **p**), plasma FITC level (**e**, **q**), urine FITC level (**f**, **r**), plasma LPS (**g**, **s**), fasting blood glucose (**h**, **t**), fasting blood insulin (**i**, **u**), HOMA-IR index (**j**, **v**), glucose tolerance (**k**, **w**), and insulin sensitivity (**l**, **x**) in C57Bl/6 (**a**–**l**) and CD-1 (ICR) mice (**m**–**x**). *n* = 5. Data are expressed as the mean ± SEM. *P < 0.05. NFD-R re-colonized with NFD group’s microbiota, NCPF-R re-colonized with NCPF group’s microbiota

The effects of chlorpyrifos-altered microbiota on obesity and glucose homeostasis were further investigated by microbiota transplantation. NFD-fed C57Bl/6 and CD-1 (ICR) mice were subjected to a microbiome depletion paradigm followed by adoptive transfer of cecal plus colonic contents collected from NFD or NCPF groups. PCA visualization demonstrated that the microbial composition of re-colonized with NFD (NFD-R) and re-colonized with NCPF (NCPF-R) mice was similar to their initial donors (Fig. 7). The taxonomical distributions of NFD-R and NCPF-R groups at phylum, family, and genus levels for the cecal samples are shown in Additional file 5: Figure S5. As shown in Fig. 8, chlorpyrifos-altered microbiota could significantly increase the percent of body weight change, epididymal fat pad weight, epididymal fat pad content of whole body weight (%), urine FITC concentration, plasma LPS concentration, fasting blood glucose, and HOMA-IR index and significantly decreased insulin sensitivity (assessed by ITT) and glucose tolerance (assessed by OGTT) in C57Bl/6 mice. Chlorpyrifos-altered microbiota could also affect these indicators in CD-1 (ICR) mice, but only urine FITC concentration, HOMA-IR index, insulin sensitivity (assessed by ITT), and glucose tolerance (assessed by OGTT) were significantly changed. All these data indicated that gut microbiota was one of the main reasons for chlorpyrifos-induced obesity and IR in mice.

## Discussion

Increasing evidence has shown that the use of global pesticides has increased the risk of developing obesity and T2D [34, 35]; however, the mechanisms are not well understood, and to the best of our knowledge, no report has focused on the mechanisms underlying the effects of pesticides on the gut microbiota and obesity. Gut microbiota, which consists of a large number of bacteria (10-fold more than the total number of human cells), shapes many important physiological and metabolic processes in the body [36, 37]. The gut microbiota in the human body is not simply a parasite, but rather engages in a symbiotic relationship. In addition to absorbing nutrients in food residues in the host gut, intestinal bacteria can provide bioactive substances and has important effects on the host in various pathophysiological aspects including immunity, body condition, and body weight [38, 39]. Therefore, pesticide-induced obesity may occur through its effects on the gut microbiota [40]. Here, we report for the first time that the OP pesticide chlorpyrifos may increase LPS levels in the body by enhancing intestinal permeability to induce chronic inflammation, and ultimately leading to IR and obesity. And the chlorpyrifos-altered microbiota could affect the occurrence of obesity and impaired glucose homeostasis.

It has been well documented that both genetic and dietary factors have significant impacts on obesity. To comprehensively study the effects of chlorpyrifos on individuals with different genetic background and dietary habits, two dietary patterns (high fat and normal fat) and two strains of mice (C57Bl/6 and CD-1 (ICR)) were chosen to determine the pathogenesis and consequences of chlorpyrifos-induced obesity. The results showed that a HFD had significant effects on body composition and IR in both C57Bl/6 and CD-1 (ICR) mice, which are consistent with previous studies [7]. Chlorpyrifos had significant effects on body weight and percent of body weight change in mice fed a NFD, but not in those fed a HFD, suggesting that the influence of chlorpyrifos on body weight is related to dietary habits. The results from measuring absolute epididymal weight and epididymal fat pad content of whole body weight (%) indicated that chlorpyrifos significantly increased them in the body, which were not related to dietary patterns or genetic background. Together, these data suggest that chlorpyrifos-induced weight gain was largely attributed to the increased fat weight. Because visceral fat is closely related to the complications of obesity, these results indicate that the harmful effects of chlorpyrifos may extend beyond weight gain. We found that chlorpyrifos treatment resulted in significant weight gain and obesity status in both C57Bl/6 and CD-1 (ICR) NFD-fed mice; however, in HFD-fed mice, although chlorpyrifos did not significantly affect weight gain relative to their control group, their fat mass was increased and the obesity-related symptoms worsened. This notion was supported by the results of the insulin sensitivity tests. Chlorpyrifos induced an increase in IR in NFD-fed mice, as well as in HFD-fed mice, even though weight gain was not significantly affected by chlorpyrifos. Insulin is a key hormone in the body that is responsible for regulating the metabolism of carbohydrates, lipids, and proteins. IR causes reduced sensitivity to insulin action in the cells of fat, liver, and skeletal muscle so that normal levels of insulin fail to efficiently reduce blood glucose, leading to hyperglycemia. IR is believed to be a major factor in the pathogenic mechanism of metabolic syndrome and T2D development. While the generation of IR is still not completely understood, factors such as genetic background, diet, and low-grade inflammation are known to be involved. In this study, genetic background and diet were the control variables; therefore, chlorpyrifos-induced IR is more likely to be mediated by the low-grade inflammation pathway.

LPS is an integral component of the outer membranes of Gram-negative bacteria, and chronic exposure to low-dose LPS can induce low-grade inflammation [41, 42]. A HFD could increase intestinal permeability and LPS, thereby leading to low-grade chronic systemic inflammation [13, 28]. In this study, HFD groups were found to have higher intestinal permeability, LPS concentration, and inflammation markers than NFD groups. A previous study using in vitro model based on an enterocyte cell line showed that chlorpyrifos can interfere with tight junctions, altering the barrier integrity and increasing intestinal permeability [26]. Based on these results, we speculate that chlorpyrifos impairs the integrity of intestinal cells to result in intestinal inflammation, which allows increased LPS entry into the body culminating in endotoxemia. This speculation was supported by the results that chlorpyrifos could reduce the mRNA expression of tight junction proteins in the ileum and colon, and further supported by the results in intestinal permeability and LPS assays. Next, the activity of MPO and the fecal lipocaline-2 level was determined, and the results showed that chlorpyrifos treatment caused higher MPO activity and fecal lipocaline-2 level in NFD-fed mice. Both MPO activity and fecal lipocaline-2 level were important indicators of gut inflammation. In addition, the results of inflammatory mediator expression (TNF-α, PAI-1, IL-1 β, and MCP-1) in the ileum and colon further confirmed that chlorpyrifos treatment could lead to gut inflammation in NFD mice. Previous studies have shown that gut inflammation is often associated with systemic low-grade inflammation [43]. The expression of LPS receptor TLR-4 and related inflammatory mediators in the liver and fat tissues and plasma proinflammatory cytokines concentrations were further determined and found that these mediators could be increased by chlorpyrifos treatment. In previous studies in human and animals, IR individuals are often found to have low-grade inflammation and increased levels of inflammatory mediators such as TNF-α, PAI-1, IL-1 β, and MCP-1 [44–46]. These inflammatory mediators can interfere with the insulin signaling pathway to cause IR. Together with our results, it can be suggested that chlorpyrifos may increase the entry of LPS into the body by promoting intestinal permeability to induce low-grade inflammation eventually leading to IR and obesity.

Previous studies have shown that dietary pattern could significantly impact the gut microbiota composition [47]. In this study, HFD decreased *Bacteroidetes* and increased *Firmicutes*, which are typical HFD-induced change in gut microbiota and related to obesity [29, 48]. On the other hand, exogenous compounds that affect gut microbiota composition and alter composition can directly impact the host metabolism [10, 32, 49]. Thus, in this study, the effects of chlorpyrifos on the gut microbiota were investigated, and the results showed that the gut microbiota in mice fed HFD was not significantly affected by chlorpyrifos treatment. A possible explanation is that the gut microbiota can be affected by HFD dominantly [48, 50], compared with which the impact of chlorpyrifos was limited. Thus, it is conceivable to have observed that gut microbiota was not significantly altered by chlorpyrifos in HFD-fed mice. Many previous studies showed similar results that xenobiotics-altered microbiota could impact host’s condition [10, 32, 51]. By comparing changes in the microbiota of NFD-fed C57Bl/6 and CD-1 (ICR) mice, the core microflora that could be affected by chlorpyrifos treatment were identified, and according to previous studies, this changed microbiota composition might be the reason for the results of microbiota transplantation. The core affected microflora including increased *Proteobacteria* phyla and decreased *Bacteroidetes* phyla. Of particular note, increased LPS-bearing *Proteobacteria* and decreased *Bacteroidetes* phyla are reportedly associated with obesity [52, 53]. In addition, in the analysis of individual bacteria species, we found 31 OTUs that were affected by chlorpyrifos.

To investigate the effects of chlorpyrifos-altered microbiota, half of the mice in NCPF group were treated with antibiotics after 8-week chlorpyrifos treatment. The results showed that chlorpyrifos-led obesity and IR were completely restored by antibiotic treatment for 4 weeks, suggesting that gut bacteria were involved in chlorpyrifos-induced obesity and IR. In addition, the results of microbiota transplantation experiment using NCPF and NFD groups’ microbiota showed that chlorpyrifos-altered microbiota could also induce obesity and IR, especially in NFD-fed C57Bl/6 mice. The above results suggested that chlorpyrifos-altered microbiota should be one of the reasons for the increased percent of fat weight and impaired insulin sensitivity in mice. Thus, chlorpyrifos not only has direct effects on the body, but also negatively impacts glucose homeostasis and obesity by altering gut microbiota composition.

## Conclusion

In this study, we found that chlorpyrifos impaired intestinal integrity to promote more LPS entry into the body resulting in low-grade inflammation, which ultimately led to IR and obesity. During this process, obese mice had more severe symptoms, while healthy mice fed NFD developed IR and obesity. Similar results were observed in mice with different genetic backgrounds, which indicate that this process may not be dependent upon genetic background. In addition, the results of antibiotic treatment and microbiota transplantation experiments showed that chlorpyrifos-altered microbiota were involved in chlorpyrifos-induced obesity and IR. Together, our results suggest that chlorpyrifos may promote metabolic syndrome by altering gut and gut microbiota. These results should be addressed with regard to pesticide safety evaluations in future studies.

## Methods

### Materials

Chlorpyrifos (98%, technical grade) was obtained from the Institute for the Control of Agrichemicals, Ministry of Agriculture of China. Corn oil, glucose, insulin, and fluorescein isothiocyanate (FITC)-labeled dextran 4 kDa (FD4) were purchased from Sigma-Aldrich (St. Louis, MO, USA). NFD (10% lipids) and HFD (60% lipids) were made by TROPHIC Animal Feed High-tech Co., Ltd. (Nantong, Jiangsu, China). Diets were maintained at − 80 °C until administration.

### Animals

Animal experiments were approved and performed in accordance with the guidelines of Institutional Animal Care and Use Committee of China Agricultural University (approval no. CAU20160302-3). Three-week-old male C57Bl/6 and CD-1 (ICR) mice were purchased from Beijing Vital River Laboratory Animal Technology Co., Ltd. Mice were housed in standard cages in a specific pathogen-free facility with a 12:12-h light:dark photoperiod. After 7 days of acclimation to a NFD, the mice were randomly divided into five groups (*n* = 8 for each group): NFD with chlorpyrifos administered by gavage at daily doses of 5 mg/kg (dissolved in corn oil), assigned as NCPF; NFD with chlorpyrifos administered by gavage at daily doses of 5 mg/kg (dissolved in corn oil), and with 1.0 g/L ampicillin and 0.5 g/L neomycin in drinking water (beginning at week 8, last for 4 weeks) [28], assigned as NCPF-A; NFD with corn oil as vehicle, assigned as NFD; HFD with chlorpyrifos administered by gavage at daily doses of 5 mg/kg (dissolved in corn oil), assigned as HCPF; and HFD with corn oil as vehicle, assigned as HFD (the composition of NFD and HFD was shown in Additional file 6: Table S1). After 12 weeks of treatment, mice were euthanized, and blood was collected and centrifuged to obtain plasma. Colon length and epididymal adipose tissue pads weight were measured. Liver, epididymal adipose tissue pads, and cecal contents were collected, immediately snap-frozen in liquid nitrogen, and stored at − 80 °C.

Three-week-old male C57Bl/6 and CD-1 (ICR) mice were acclimated to a normal-fat diet and then randomly divided into two groups: re-colonized with NFD group’s microbiota, assigned as NFD-R; re-colonized with NCPF (normal-fat diet with chlorpyrifos) group’s microbiota, assigned as NCPF-R. Mice were given a cocktail of antibiotic (0.25 mg/day ampicillin, gentamicin, metronidazole, neomycin, and 0.125 mg/day vancomycin) once daily for 12 consecutive days by gavage, and then re-colonized 72 h later via daily oral gavage of donor microbiota for 3 days. To reinforce the donor microbiota genotype, microbiota were given weekly through the study [51, 54]. Mice were fasted for 12 h and 6 h to perform OGTT (3 weeks after re-colonization) and ITT (4 weeks after re-colonization). After 5-weeks’ re-colonization, mice were euthanized via CO_2_ asphyxiation, and blood was collected and centrifuged to obtain plasma. Body weight and epididymal adipose tissue pads weight were measured.

### Food intake measurement

Groups of mice were placed in a clean cage with weighted amount of food. The weight of the remaining food was measured 24 h later with the difference viewed as food intake per 24 h. Error bars represent SEM of three measurements made 1 week apart.

### Glucose homeostasis measurements

At week 10, animals were deprived of food for 12 h and OGTT was performed after gavage with 2 g of glucose per kilogram body weight in sterile phosphate-buffered saline and blood glucose levels were measured with an Accu-Check Glucose Meter (Roche Diagnostic, Milan, Italy) at 0, 15, 30, 60, and 120 min (*n* = 8). At the end of week 11, mice were deprived of food for 6 h and ITT was performed after intraperitoneal injection of 0.5 U insulin per kilogram body weight and blood glucose concentrations were detected at 0, 15, 30, 60, and 120 min (*n* = 8).

### In vivo epithelial barrier permeability

After 11 weeks of treatment, mice were fasted for 12 h and administrated with 600 mg/kg body weight of 80 mg/mL FD4. Blood and urine were collected before (as background, T0) and after the gavage (2 h), and plasma and urine fluorescence levels were estimated by fluorometric determination (excitation, 490 nm; emission, 520 nm; BIOTEK Fluorescence Spectrophotometer, Winooski, VT, USA).

### Biochemical assay

The enzymatic activity of myeloperoxidase (MPO) was determined (*n* = 8) using a commercial myeloperoxidase assay kit (Nanjing Jiancheng Bioengineering Institute, Jiangsu China). Plasma LPS, insulin, TNF-α, MCP-1, IL-1 β, PAI-1, fecal LPS, and lipocaline-2 quantification were performed (*n* = 8) using commercial enzyme-linked immunosorbent assay (ELISA) kits (Nanjing Jiancheng Bioengineering Institute).

### Gene expression analysis by qPCR

Total RNA was isolated from tissues (*n* = 5) using a TransZol™ UP kit (Transgen Biotech, Beijing, China) as specified by the manufacturer. RNA was converted to cDNA using TransCript All-in-One First-Strand cDNA Synthesis SuperMix (Transgen Biotech). qPCR was performed in triplicate using TransStart Top Green qPCR SuperMix and LineGene9600 Plus (Bioer, Hangzhou, China). Primer sequences (purchased from Sunbiotech Co., Ltd., Beijing, China) for the targeted genes were as follows: TLR4, forward, GCAGAAAATGCCAGGATGATG, reverse, AACTACCTCTATGCAGGGATTCA-AG; tumor necrosis factor-alpha (TNF-α), forward, GACCCTCACACTCAGATCA-TCTTCT, reverse, CCACTTGGTGGTTT-GCTACGA; monocyte chemoattractant protein-1 (MCP-1), forward, GGCTCAGCC-AGATGCAGTTAA, reverse, CCTACTCATTGGGATCATCTTGCT; interleukin-1 beta (IL-1 β), forward, TCGCTCAGGG-TCACAAGAAA, reverse, CATCAGAGG-CAAGGAGGAAAAC; plasminogen activator inhibitor-1 (PAI-1), forward, ACAGCCTTTGTCATCTCAGCC, reverse, CCGAACCACAAAGAGAAAGGA; occludin, forward, CGGCTATGGAGGCTATGGCTATG, reverse, ATGAACCCCA-GGACAATGGC; ZO-1, forward, ATCCCAAATAAGAACAGAGC, reverse, GGC-GTTACATCTAATAAAGC; claudin 1, forward, TTGTTTGCAGAGACCCCATC-AC, reverse, GGAGTAAATCTTCCACTGGGGC and ribosomal protein L19 (RPL19) (internal control), forward, GAAGGTCAAAGGGAATGTGTTCA, reverse, CCTTGTCTGC-CTTCAGCTTGT.

### Gut microbiota analysis

DNA was extracted from cecal feces using the QIAamp DNA Stool Kit (Qiagen, Gaithersburg, MD, USA) according to the manufacturer’s protocols (*n* = 5 for HFD of C57Bl/6 mice, *n* = 6 for HCPF of C57Bl/6 and NFD of CD-1 (ICR) mice, *n* = 7 for other mice). Total bacterial abundance was assessed via the standard curve with plasmid DNA as template. The 16S rRNA genes, hypervariable region V4–V5, were amplified by PCR (2 min at 95 °C, followed by 25 cycles of 30 s at 95 °C, 30 s at 55 °C, 30 s at 72 °C, and 5 min at 72 °C) using the special primers (515F: 5′-NNNNNNNN-GTGCCAGCMG-CCGCGG-3′; 907R: 5′-CCGTCAATTCMTTT-RAGTTT-3′; “N” indicates the nucleotides of the barcode sequence). PCR reactions were performed in triplicate in a 20-μL mixture (4 μL 5× FastPfu Buffer, 2 μL of 2.5 mM dNTPs, 0.8 μL of 5 μM of each primer, 0.4 μL FastPfu Polymerase, and 10 ng template DNA). Then, the PCR products were purified with the AxyPrep DNA Gel Extraction Kit (Axygen, Union City, CA, USA) and quantified by QuantiFluor™-ST (Promega, Madison, WI, USA). Purified amplicons were pooled on an Illumina MiSeq platform according to standard protocols. QIIME (version 1.17) was used to select raw fastq files, and the denoising criteria comprised the following: (1) The 250 base pair (bp) reads were truncated at any site receiving an average quality score < 20 over a 10-bp sliding window, discarding the truncated reads that were shorter than 50 bp. (2) Exact barcode matching, two nucleotide mismatch in primer matching, reads containing ambiguous characters were removed. (3) Only sequences that overlapped longer than 10 bp were assembled according to their overlap sequence. High-quality reads were selected and clustered into Operational Units (OTUs) based on 97% similarity cutoff using UPARSE (version 7.1 http://drive5.com/uparse/). UCHIME was used to identify and remove chimeric sequences. All 16S rRNA gene sequencing reads data has been deposited to the National Center for Biotechnology Information’s Sequence Read Archive under accession number SRP100961.

### Statistical analysis

Data are expressed as mean ± standard error of the mean (SEM). Statistical analyses were performed using SPSS 20.0 (IBM Corp., Armonk, NY, USA). Datasets that involved four groups were performed using one-way analysis of variance with a post hoc Bonferroni multiple comparison test. Independent samples *t* test (two-tailed) was used to compare microbial community structures between NFD and NCPF. *P* values less than 0.05 were considered statistically significant.

## Additional files

Additional file 1: **Figure S1.** Effects of chlorpyrifos administration on food intake (a and e), colon length (b and f), fecal bacteria amount (c and g), and fecal LPS levels (d and h) in C57Bl/6 (a–d) and CD-1 (ICR) mice (e–h). Data are expressed as the mean ± SEM. *P < 0.05 versus NFD group; # *P* < 0.05 versus HFD group. NFD, normal-fat diet; NCPF, normal-fat diet + chlorpyrifos; HFD, high-fat diet; HCPF, high-fat diet + chlorpyrifos. (DOCX 197 kb)

Additional file 2: **Figure S2.** Effects of chlorpyrifos treatment on the concentration of proinflammatory cytokines in plasma in C57Bl/6 (a–d) and CD-1 (ICR) mice (e–h). Data are expressed as the mean ± SEM. *P < 0.05 versus NFD group; # *P* < 0.05 versus HFD group. NFD, normal-fat diet; NCPF, normal-fat diet + chlorpyrifos; HFD, high-fat diet; HCPF, high-fat diet + chlorpyrifos. (DOCX 202 kb)

Additional file 3: **Figure S3.** Microbiota membership for cecal samples of C57Bl/6 (a, c, and e) and CD-1(ICR) (b, d, and f) mice. Box plots depicting the taxonomic distribution within NFD, NCPF, HFD, and HCPF cecal samples at the phylum, family, and genus levels. NFD, normal-fat diet; NCPF, normal fat-diet + chlorpyrifos; HFD, high-fat diet; HCPF, high-fat diet + chlorpyrifos. (DOCX 457 kb)

Additional file 4: **Figure S4.** Heatmap showing the abundance of OTUs significantly altered by chlorpyrifos (*P* < 0.05), blue and red for underrepresented and overrepresented. (a) C57Bl/6 mice fed with NFD. (b) C57Bl/6 mice fed with HFD. (c) CD-1(ICR) mice fed with NFD. (d) CD-1(ICR) mice fed with HFD. NFD, normal-fat diet; NCPF, normal-fat diet + chlorpyrifos; HFD, high-fat diet; HCPF, high-fat diet + chlorpyrifos. (DOCX 969 kb)

Additional file 5: **Figure S5.** Microbiota membership for cecal samples of C57Bl/6 (a, c, and e) and CD-1(ICR) (b, d, and f) mice. Box plots depicting the taxonomic distribution within NFD-R and NCPF-R cecal samples at the phylum, family, and genus levels. NFD-R, re-colonized with NFD group’s microbiota; NCPF-R, re-colonized with NCPF group’s microbiota. (DOCX 391 kb)

Additional file 6: **Table S1.** NFD and HFD composition. (DOCX 18 kb)
