# Supplementary material for: Organophosphorus pesticide chlorpyrifos intake promotes obesity and insulin resistance through impacting gut and gut microbiota
Source: Microbiome. 2019 Feb 11;7:19. doi: 10.1186/s40168-019-0635-4 (PMC6371608; doi:10.1186/s40168-019-0635-4)
Supplement: Supplementary file 1 — Figure S1. Effects of chlorpyrifos administration on food intake (a and e), colon length (b and f), fecal bacteria amount (c and g), and fecal LPS levels (d and h) in C57Bl/6 (a–d) and CD-1 (ICR) mice (e–h). Data are expressed as the mean ± SEM. *P < 0.05 versus NFD group; # P < 0.05 versus HFD group. NFD, normal-fat diet; NCPF, normal-fat diet + chlorpyrifos; HFD, high-fat diet; HCPF, high-fat diet + chlorpyrifos. (DOCX 197 kb) [file 40168_2019_635_MOESM1_ESM.docx]

Additional file 1


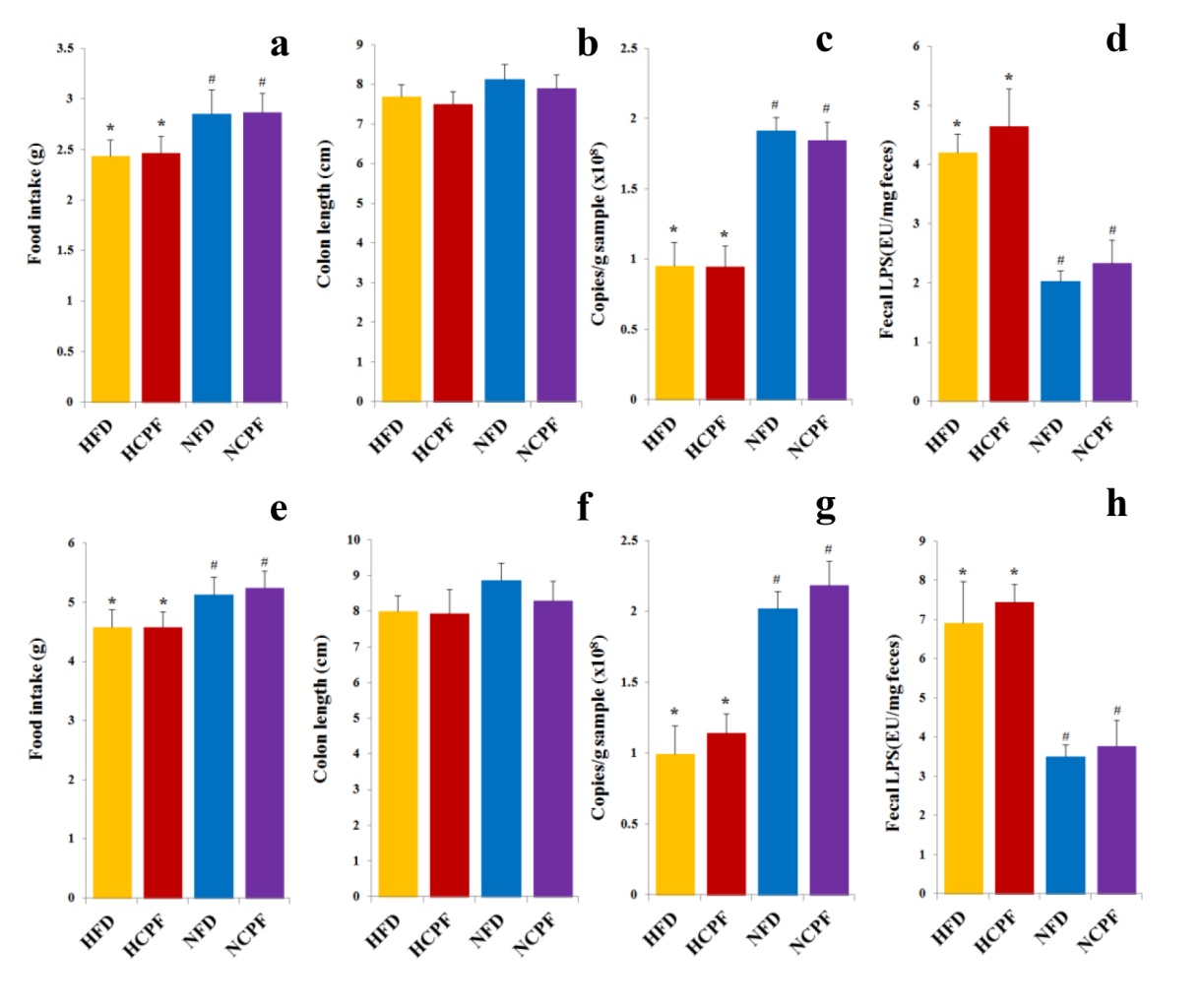


**Figure S1** Effects of chlorpyrifos administration on food intake (a and e), colon length (b and f), fecal bacteria amount (c and g) and fecal LPS levels (d and h) in C57Bl/6 (a-d) and CD-1 (ICR) mice (e-h). Data are expressed as the mean ± SEM. **P* < 0.05 versus NFD group; # *P* < 0.05 versus HFD group. NFD, normal fat diet; NCPF, normal fat diet + chlorpyrifos; HFD, high fat diet; HCPF, high fat diet + chlorpyrifos.
