# Supplementary material for: Organophosphorus pesticide chlorpyrifos intake promotes obesity and insulin resistance through impacting gut and gut microbiota
Source: Microbiome. 2019 Feb 11;7:19. doi: 10.1186/s40168-019-0635-4 (PMC6371608; doi:10.1186/s40168-019-0635-4)
Supplement: Supplementary file 2 — Figure S2. Effects of chlorpyrifos treatment on the concentration of proinflammatory cytokines in plasma in C57Bl/6 (a–d) and CD-1 (ICR) mice (e–h). Data are expressed as the mean ± SEM. *P < 0.05 versus NFD group; # P < 0.05 versus HFD group. NFD, normal-fat diet; NCPF, normal-fat diet + chlorpyrifos; HFD, high-fat diet; HCPF, high-fat diet + chlorpyrifos. (DOCX 202 kb) [file 40168_2019_635_MOESM2_ESM.docx]

Additional file 2**
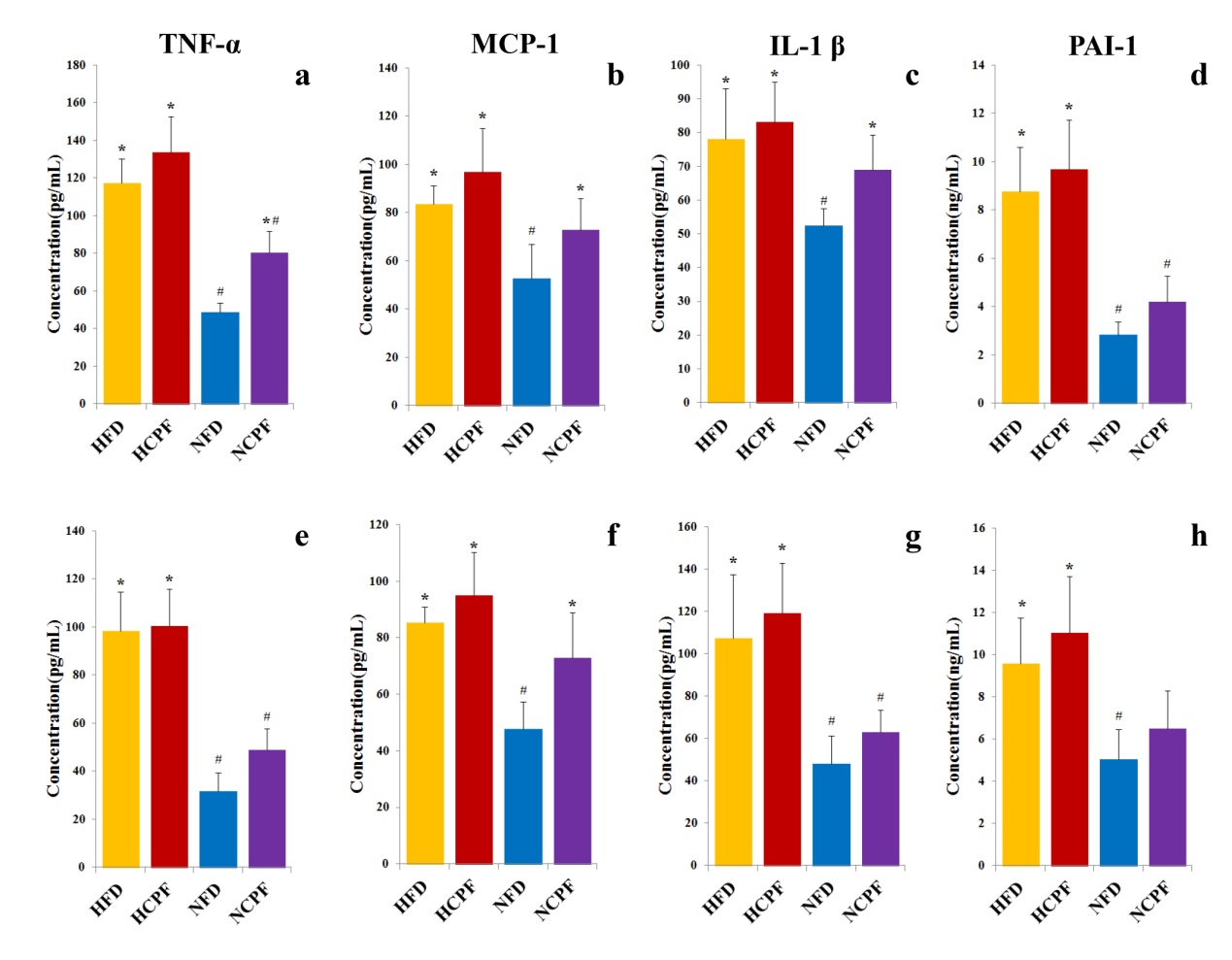
**

**Figure S2** Effects of chlorpyrifos treatment on the the concentration of proinflammatory cytokines in plasma in C57Bl/6 (a-d) and CD-1 (ICR) mice (e-h). Data are expressed as the mean ± SEM. **P* < 0.05 versus NFD group; # *P* < 0.05 versus HFD group. NFD, normal fat diet; NCPF, normal fat diet + chlorpyrifos; HFD, high fat diet; HCPF, high fat diet + chlorpyrifos.
