# Supplementary material for: Organophosphorus pesticide chlorpyrifos intake promotes obesity and insulin resistance through impacting gut and gut microbiota
Source: Microbiome. 2019 Feb 11;7:19. doi: 10.1186/s40168-019-0635-4 (PMC6371608; doi:10.1186/s40168-019-0635-4)
Supplement: Supplementary file 3 — Figure S3. Microbiota membership for cecal samples of C57Bl/6 (a, c, and e) and CD-1(ICR) (b, d, and f) mice. Box plots depicting the taxonomic distribution within NFD, NCPF, HFD, and HCPF cecal samples at the phylum, family, and genus levels. NFD, normal-fat diet; NCPF, normal fat-diet + chlorpyrifos; HFD, high-fat diet; HCPF, high-fat diet + chlorpyrifos. (DOCX 457 kb) [file 40168_2019_635_MOESM3_ESM.docx]

Additional file 3

**Phylum Level**

**b**

**b**

**a**


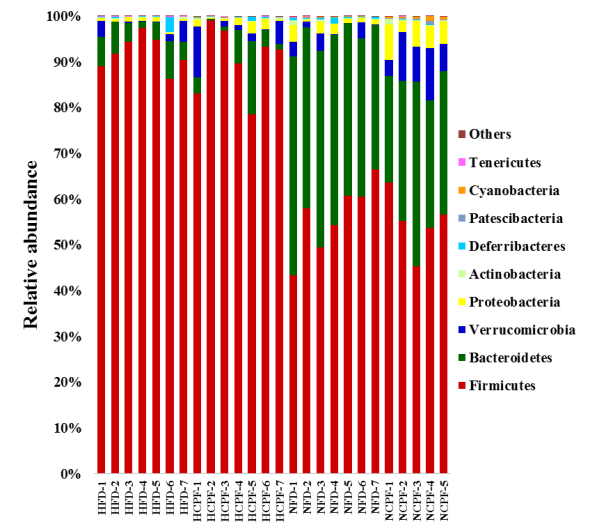

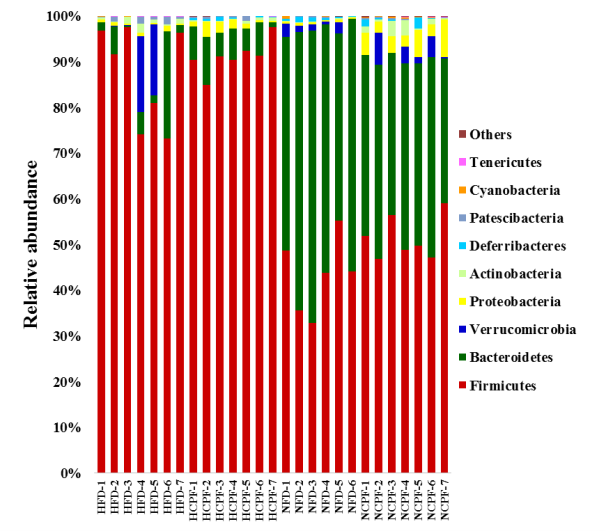


**d**

**c**

**Family Level**


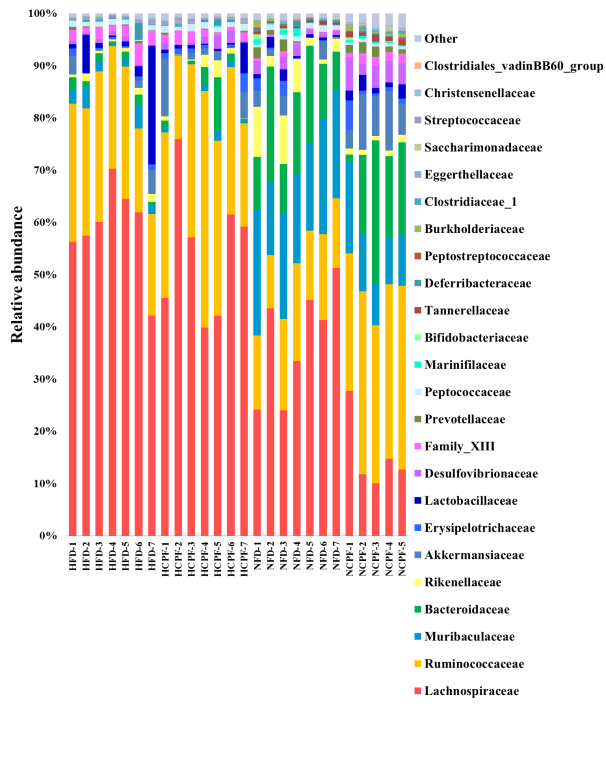

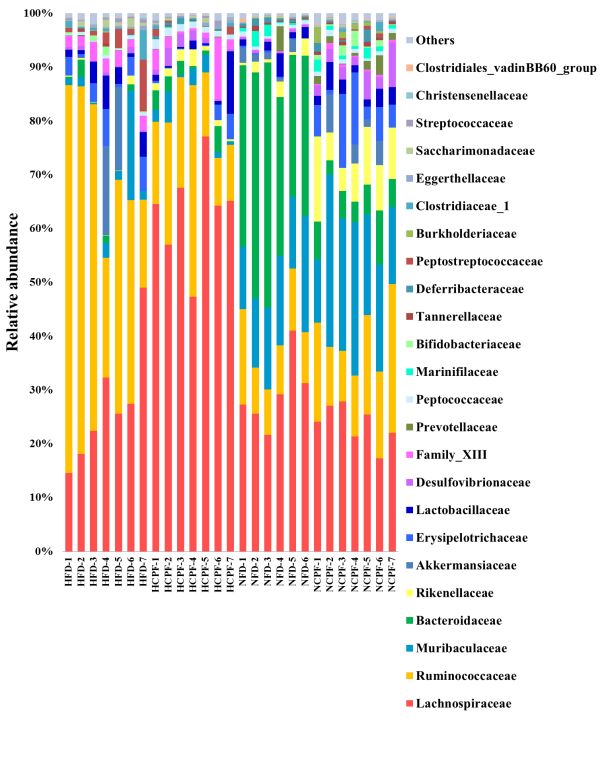


**Genus Level**

**f**

**e**


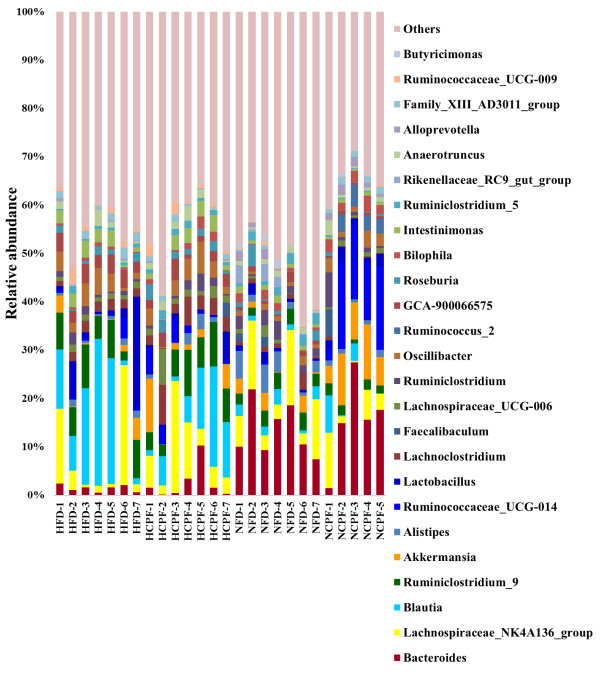

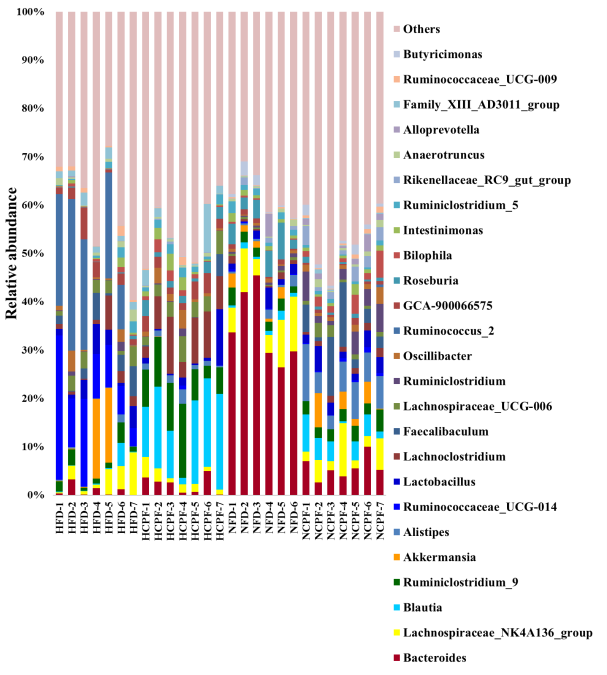


**Figure S3** Microbiota membership for cecal samples of C57Bl/6 (a, c and e) and CD-1(ICR) (b, d and f) mice. Box plots depicting the taxonomic distribution within NFD, NCPF, HFD and HCPF cecal samples at the Phylum, Family and Genus levels. NFD, normal fat diet; NCPF, normal fat diet + chlorpyrifos; HFD, high fat diet; HCPF, high fat diet + chlorpyrifos.
