# Supplementary material for: Organophosphorus pesticide chlorpyrifos intake promotes obesity and insulin resistance through impacting gut and gut microbiota
Source: Microbiome. 2019 Feb 11;7:19. doi: 10.1186/s40168-019-0635-4 (PMC6371608; doi:10.1186/s40168-019-0635-4)
Supplement: Supplementary file 4 — Figure S4. Heatmap showing the abundance of OTUs significantly altered by chlorpyrifos (P < 0.05), blue and red for underrepresented and overrepresented. (a) C57Bl/6 mice fed with NFD. (b) C57Bl/6 mice fed with HFD. (c) CD-1(ICR) mice fed with NFD. (d) CD-1(ICR) mice fed with HFD. NFD, normal-fat diet; NCPF, normal-fat diet + chlorpyrifos; HFD, high-fat diet; HCPF, high-fat diet + chlorpyrifos. (DOCX 969 kb) [file 40168_2019_635_MOESM4_ESM.docx]

Additional file 4

**a**


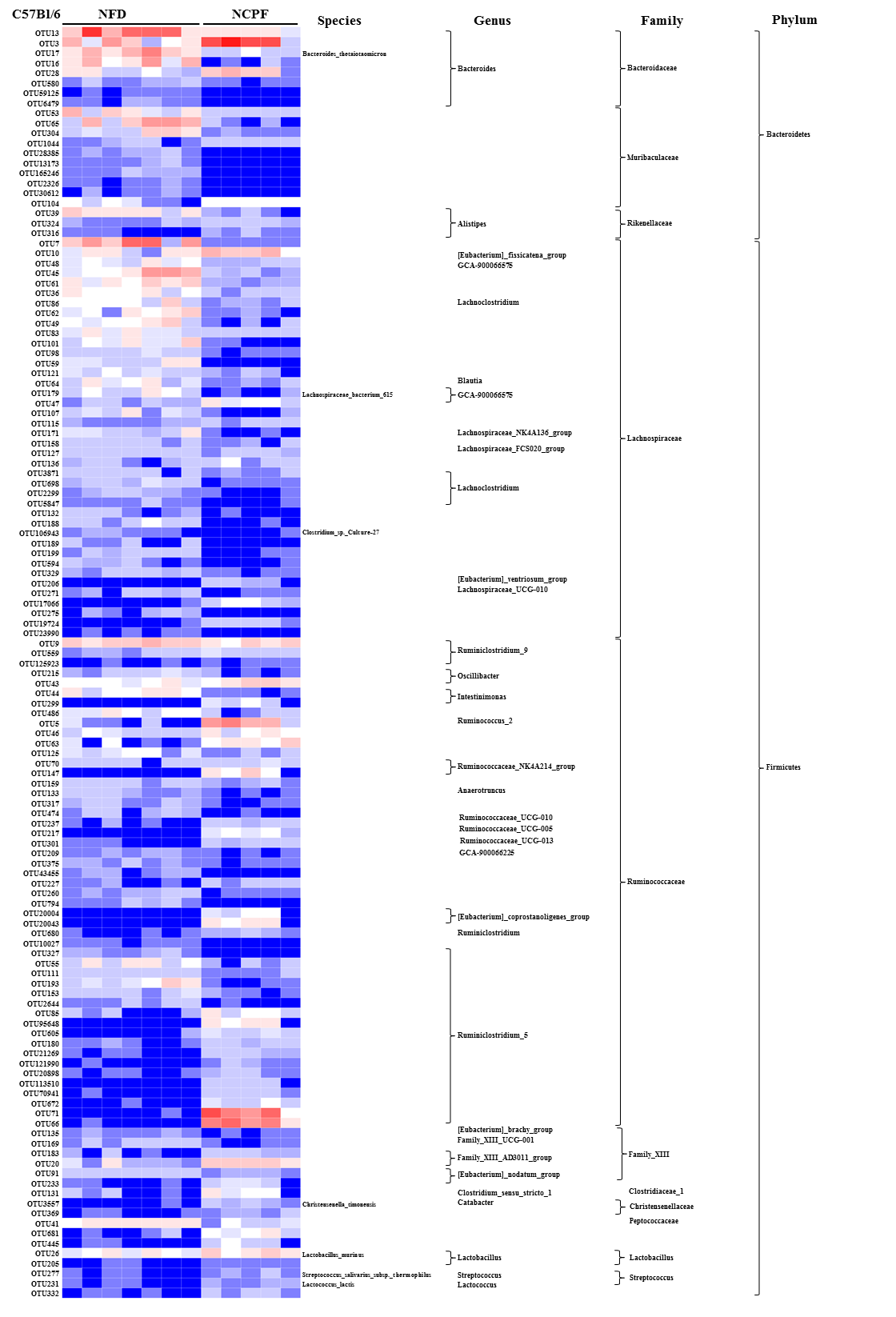


**b**


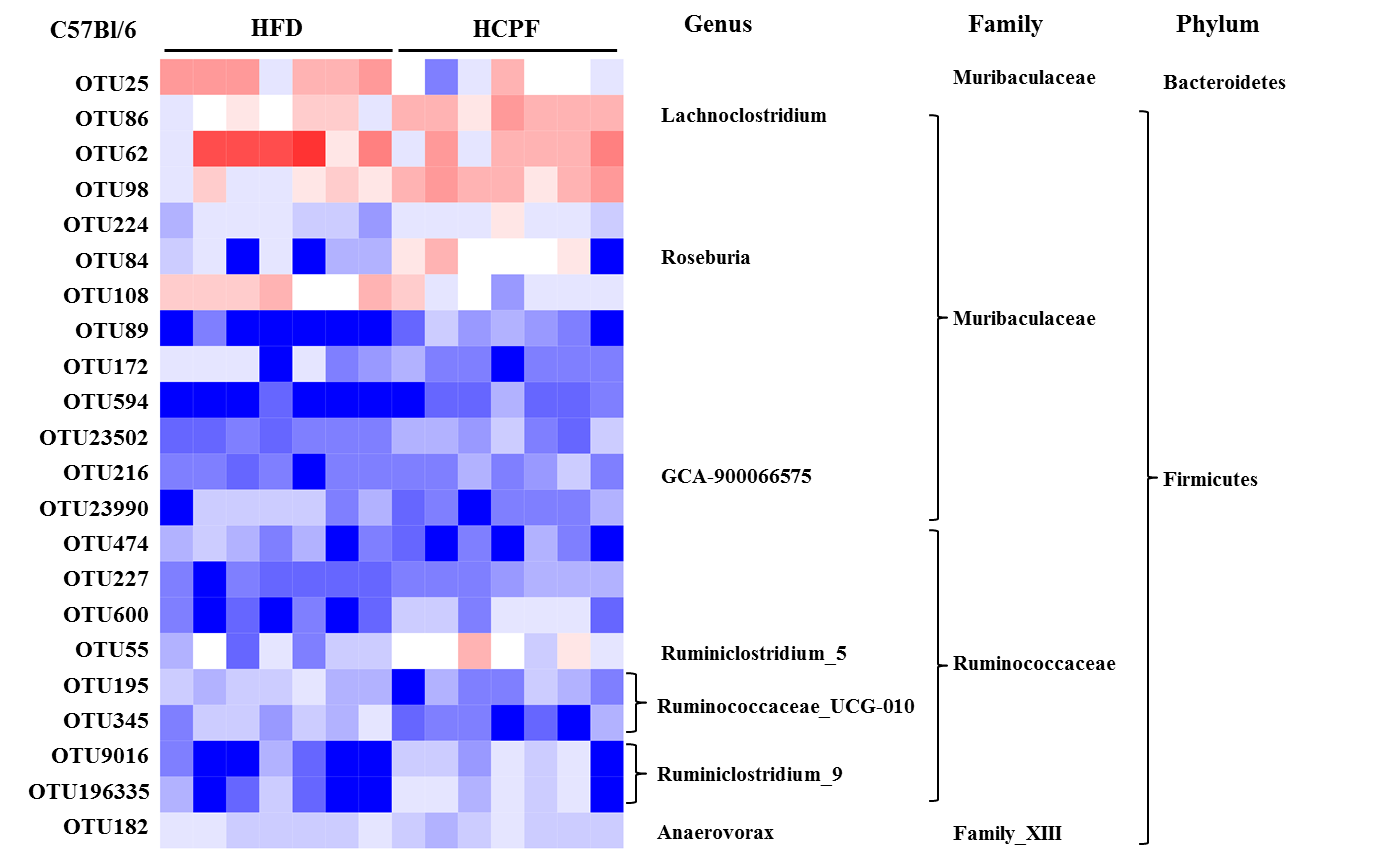


**c**


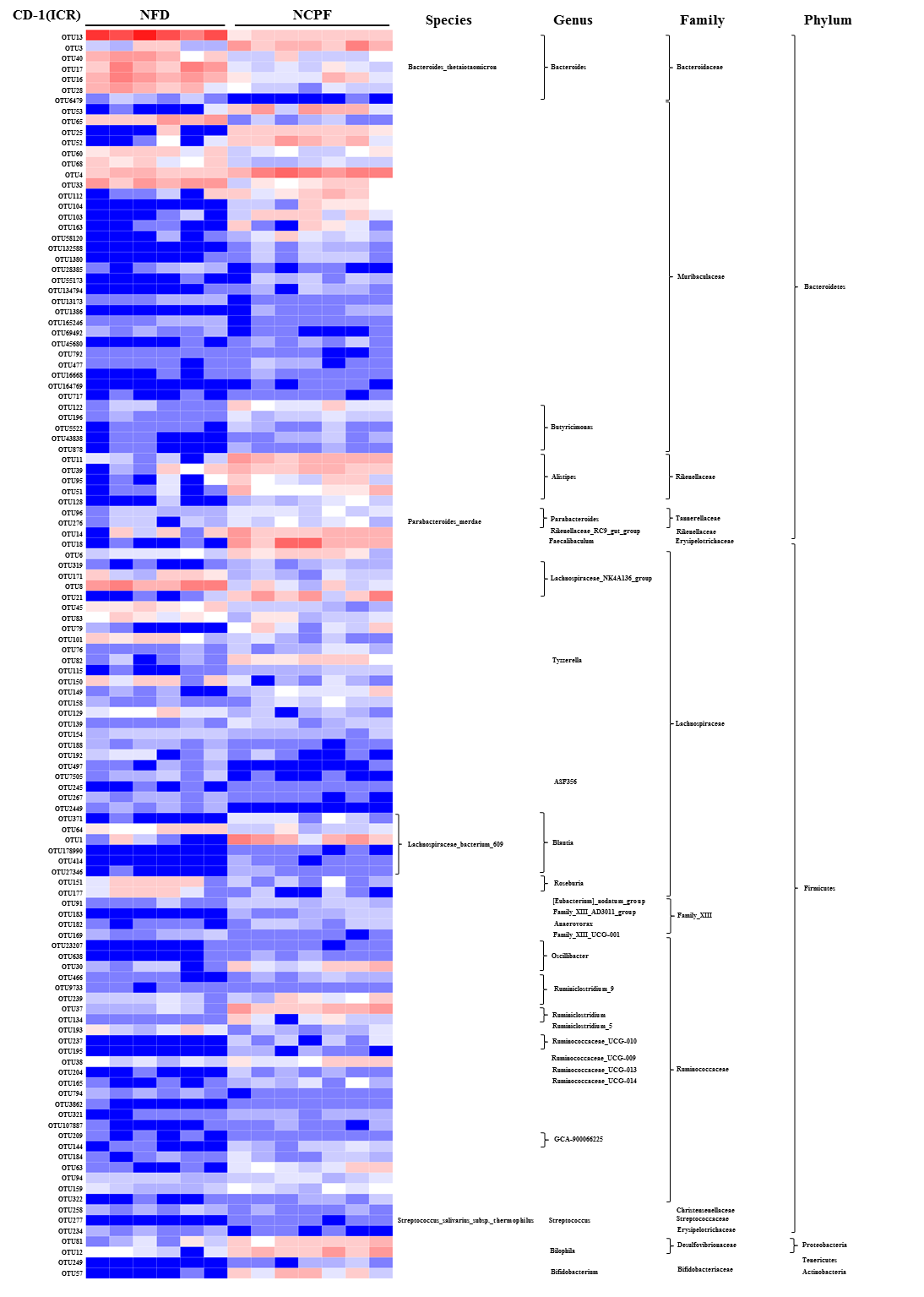


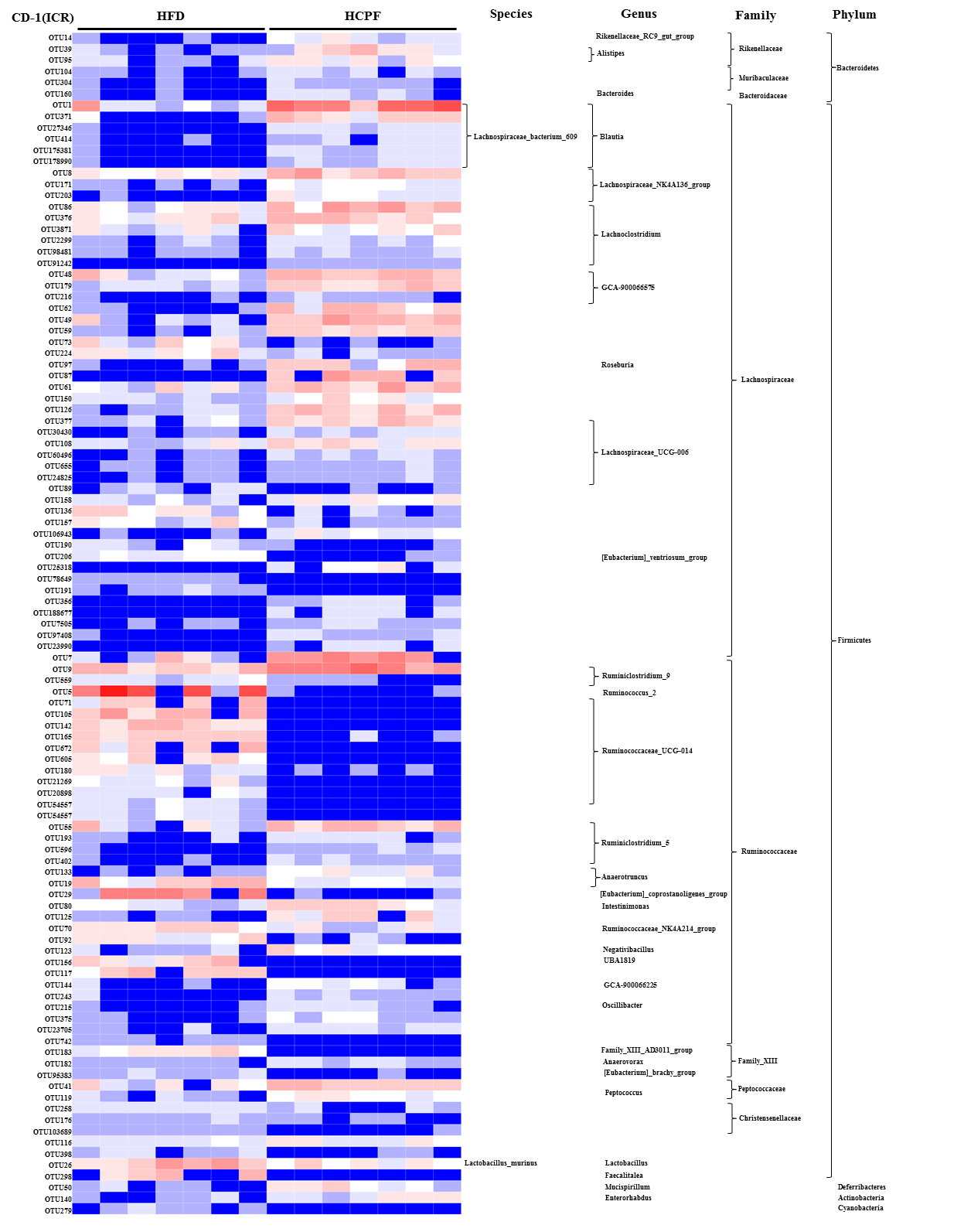


**d**

**Figure S4** Heatmap showing the abundance of OTUs significantly altered by chlorpyrifos (*P*< 0.05), blue and red for underrepresented and overrepresented. (a) C57Bl/6 mice fed with NFD. (b) C57Bl/6 mice fed with HFD. (c) CD-1(ICR) mice fed with NFD. (d) CD-1(ICR) mice fed with HFD. NFD, normal fat diet; NCPF, normal fat diet + chlorpyrifos; HFD, high fat diet; HCPF, high fat diet + chlorpyrifos.
