# Supplementary material for: Organophosphorus pesticide chlorpyrifos intake promotes obesity and insulin resistance through impacting gut and gut microbiota
Source: Microbiome. 2019 Feb 11;7:19. doi: 10.1186/s40168-019-0635-4 (PMC6371608; doi:10.1186/s40168-019-0635-4)
Supplement: Supplementary file 5 — Figure S5. Microbiota membership for cecal samples of C57Bl/6 (a, c, and e) and CD-1(ICR) (b, d, and f) mice. Box plots depicting the taxonomic distribution within NFD-R and NCPF-R cecal samples at the phylum, family, and genus levels. NFD-R, re-colonized with NFD group’s microbiota; NCPF-R, re-colonized with NCPF group’s microbiota. (DOCX 391 kb) [file 40168_2019_635_MOESM5_ESM.docx]

Additional file 5

**Phylum Level**

**b**

**a**


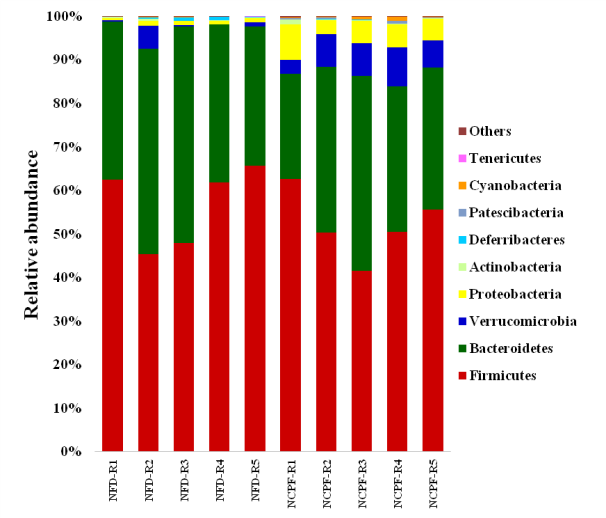

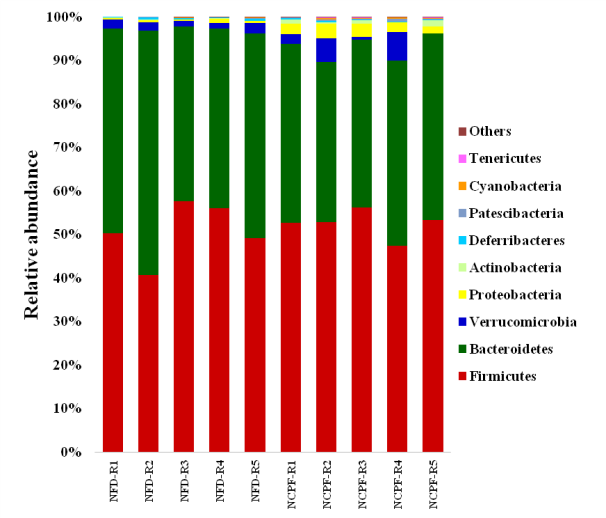


**Family Level**

**d**

**c**

**
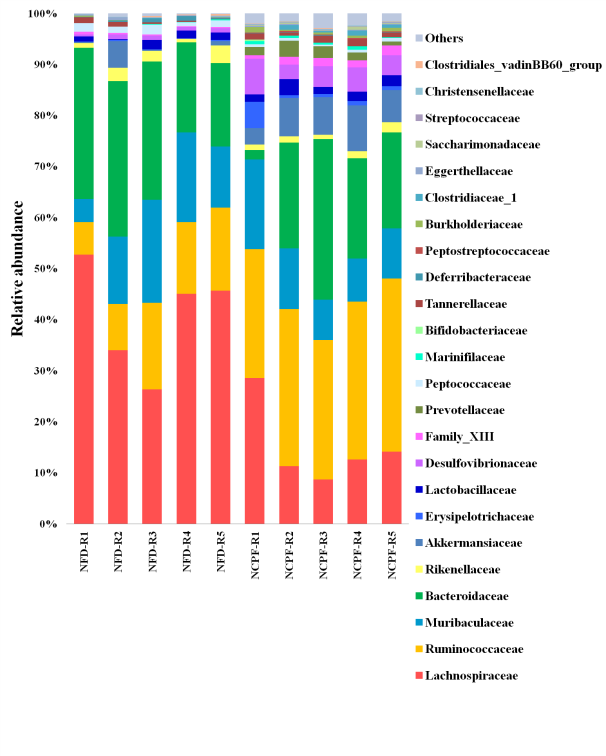
** **
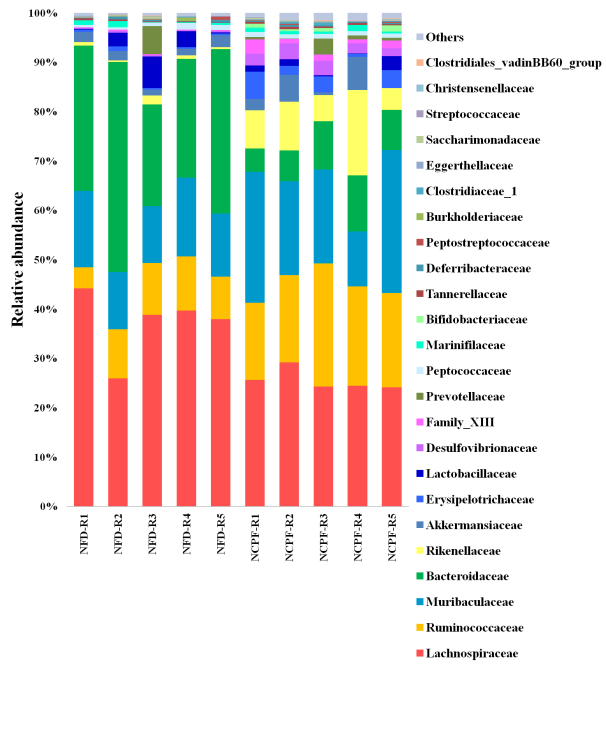
**

**Genus Level**

**e**

**f**

**
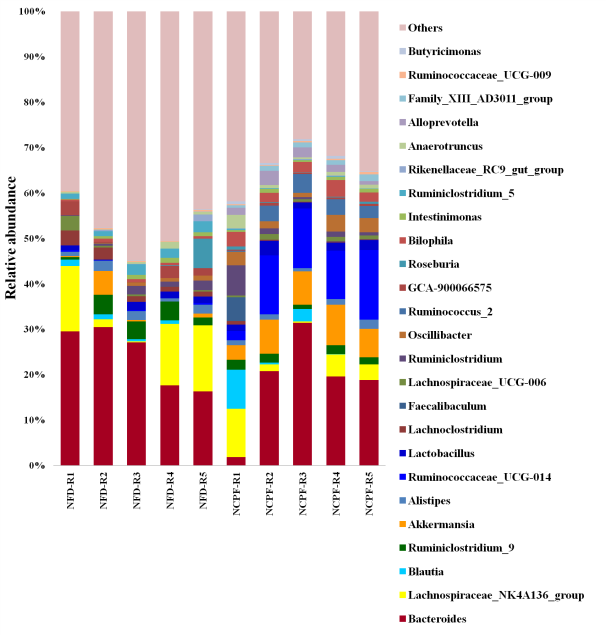

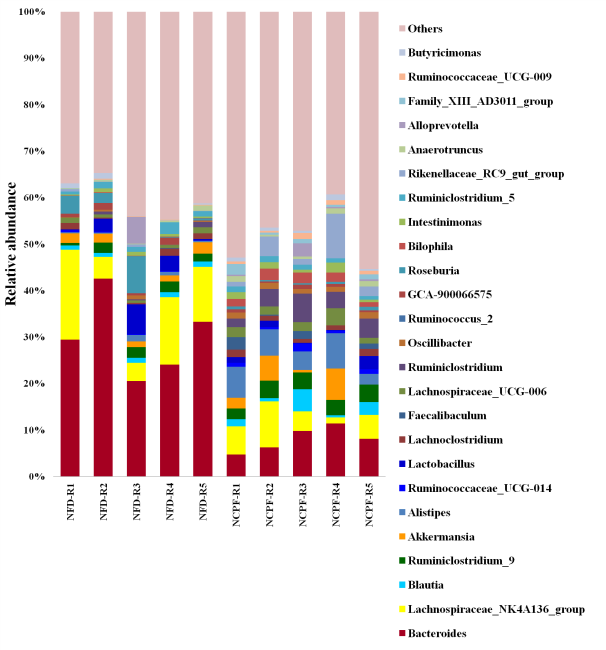
**

**Figure S5** Microbiota membership for cecal samples of C57Bl/6 (a, c and e) and CD-1(ICR) (b, d and f) mice. Box plots depicting the taxonomic distribution within NFD-R and NCPF-R cecal samples at the Phylum, Family and Genus levels. NFD-R, re-colonized with NFD group’s microbiota; NCPF-R, re-colonized with NCPF group’s microbiota.
