# Supplementary material for: Organophosphorus pesticide chlorpyrifos intake promotes obesity and insulin resistance through impacting gut and gut microbiota
Source: Microbiome. 2019 Feb 11;7:19. doi: 10.1186/s40168-019-0635-4 (PMC6371608; doi:10.1186/s40168-019-0635-4)
Supplement: Supplementary file 6 — Table S1. NFD and HFD composition. (DOCX 18 kb) [file 40168_2019_635_MOESM6_ESM.docx]

Additional file 6: **Table S1** NFD and HFD composition..

| **Material** | **NFD** | **HFD** |
| --- | --- | --- |
| Casein | 227 g/kg | 156 g/kg |
| L-Cystine | 3 g/kg | 2 g/kg |
| Corn starch | 0 g/kg | 517 g/kg |
| Dextrin | 184 g/kg | 133 g/kg |
| Sucrose | 147 g/kg | 101 g/kg |
| Soybean Oil | 0 g/kg | 25 g/kg |
| Lard | 367 g/kg | 17 g/kg |
| Mineral mix | 53 g/kg | 0.01 g/kg |
| Vitamin mix | 18 g/kg | 37 g/kg |
| TBHQ | 0.07 g/kg | 13 g/kg |
| Total | 1000 g | 1000 g |
|  |  |  |
| % as protein | 15% | 15% |
| % as carbohydrate | 25% | 75% |
| % as fat | 60% | 10% |
| **Energy** | 5.5 kcal/g | 3.8 kcal/g |
